# Suppressing MDSC Infiltration in Tumor Microenvironment Serves as an Option for Treating Ovarian Cancer Metastasis

**DOI:** 10.7150/ijbs.70013

**Published:** 2022-05-21

**Authors:** Yichen Li, Qian Zhang, Mandi Wu, Peidong Zhang, Liang Huang, Xiaolin Ai, Zhengnan Yang, Qiuhong Shen, Yiran Wang, Ping Wang, Shengtao Zhou, Ming-Liang He

**Affiliations:** 1 Department of Biomedical Sciences, JCC College of Veterinary Medicine and Life Science, City University of Hong Kong, Hong Kong SAR, China.; 2 CityU Shenzhen Research Institute, Shenzhen, China.; 3 Department of Obstetrics and Gynecology, Key Laboratory of Birth Defects and Related Diseases of Women and Children of MOE and State Key Laboratory of Biotherapy, West China Second Hospital, Sichuan University and Collaborative Innovation Center, Chengdu, 610041, Sichuan, P. R. China; 4 The State Key Laboratory of Biotherapy, West China Hospital, Sichuan University, Chengdu 610041, Sichuan, P. R. China; 5 Department of Critical Care Medicine, West China Hospital, Sichuan University, Chengdu 610041, Sichuan, P. R. China

**Keywords:** Ovarian cancer, LncRNA, PPIP5K2, Golgi complex, Complement C5, MDSC

## Abstract

It is still a big puzzle how ovarian cancer cells and the tumor microenvironment (TME) attract lymphocytes infiltration for facilitating metastasis, a leading cause of death from gynecological malignancies. Using genome-wide LncRNA microarray assay, here we report that a LncRNA associated with ovarian cancer metastasis (LncOVM) is highly correlated with poor prognosis and survival. LncOVM interacts with and stabilizes PPIP5K2 by suppressing ubiquitinated degradation to promote complement C5 secretion from ovarian cancer cells. The TME-enriched complement C5 attracts myeloid-derived suppressor cells (MDSCs) infiltration in TME to facilitate metastasis. Knockdown of LncOVM or PPIP5K2 inhibits tumor progression in xenograft models. Application of C5aR antibody or inhibitor (CCX168) inhibits MDSC recruitment and restores the suppression of tumorigenesis and metastasis *in vivo*. Our study reveals that suppression of ovarian cancer metastasis can be achieved by targeting MDSC infiltration in TME through disrupting LncOVM-PPIP5K2-complement axis, providing an option for treating ovarian cancer patients.

## Introduction

Metastasis is the main cause for the high mortality of ovarian cancer patients 1. Due to the asymptomatic nature and lack of effective screening options in early stage, over 75% of ovarian cancer patients are diagnosed at advanced stages (stage III/IV) with metastasis 2, 3. Accumulating evidence shows that the tumor microenvironment (TME) plays a crucial role in the primary tumor progression, imitation and organ-specific metastasis, and therapeutic response 4. Tumor cells and surrounding stromal cells as well as their secreted factors attract and teach lymphocytes from antitumor to promote tumor progression and metastasis 4-6. Particularly, it is reported that myeloid-derived suppressor cells (MDSCs) serve as immunosuppressors in the tumor microenvironment 5. MDSCs, a subpopulation of immunoregulatory immature myeloid cells, expand in pathological situations such as cancer, as a result of an altered hematopoiesis 5, 7. They exert immunoinhibitory activity in ovarian cancers by multiple ways, such as suppressing anti-tumor CD8^+^ T-cell, enhancing cancer cell stemness to increase metastatic potential, and so on 5. Recent studies showed that the complement system is involved in the tumor progression and metastasis in the tumor microenvironment, in which T cells and MDSCs are major players 8-11. In addition, some cytokines and chemokines secreted from omental adipocytes 12, such as interleukin (IL)-6 13, IL-8 14, 15, TIMP metallopeptidase inhibitor 1 (TIMP1) 16, 17, are reported to involve in lymphocyte infiltration in ovarian cancer metastasis 18, 19.

It has been shown that long noncoding RNAs (LncRNA), a subclass of noncoding RNA with over 200 nucleotides, are involved in regulating cytokines/chemokines production and secretion, and lymphocytes infiltration during cancer metastasis^20^, ^21^. Identification of metastasis-associated LncRNA would not only help us to better understand the regulation of LncRNA-cytokine/chemokine-lymphocyte infiltration-metastasis axis; more importantly, they may serve as potential early diagnosis markers and even as therapeutic targets for ovarian cancer prevention and therapy.

Inositol hexakisphosphate and diphosphoinositol-pentakisphosphate kinase 2 (PPIP5K2), as a bifunctional inositol kinase, it regulates a variety of cellular processes, including apoptosis, vesicle trafficking, cytoskeletal dynamics, exocytosis, and so on^22^-^26^. As for the function of PPIP5K2 in cancers, it has been reported that the PPIP5K2 was highly expressed in colorectal cancer (CRC) and associated with a poor prognosis of CRC patients 27-30. However, no study has reported its function and mechanism in ovarian cancer to our knowledge.

In this study, we employed genome-wide LncRNA array assay and identified a metastasis-associated LncRNA, LncOVM, which interacts with and stabilizes PPIP5K2 to regulate complement secretion for inducing MDSCs into TME. We show that targeting LncOVM-PPIP5K2-complement axis prevents ovarian cancer metastasis and significantly extends the survival of tumor-loaded animals.

## Results

### LncOVM correlates with metastasis and poor prognosis in ovarian cancer patients

MDSCs are reported to involve in metastasis of many other cancer types 11, 31, 32, which may serve as immunosuppressors in the tumor microenvironment 32, 33. In clinic practice, the increased MDSCs were reported in the peripheral blood and ascites of ovarian cancer patients 34. In an initial study, we confirmed the severe metastasis in the ovarian tumor-bearing mice (Fig.S1A). Consistent with another study 35, MDSCs were found to infiltrate into the tumor microenvironment and ovarian tumors (Fig.S1B & S1C). MDSCs largely infiltrated in TME, suggesting their importance in metastasis.

**Figure 1:**
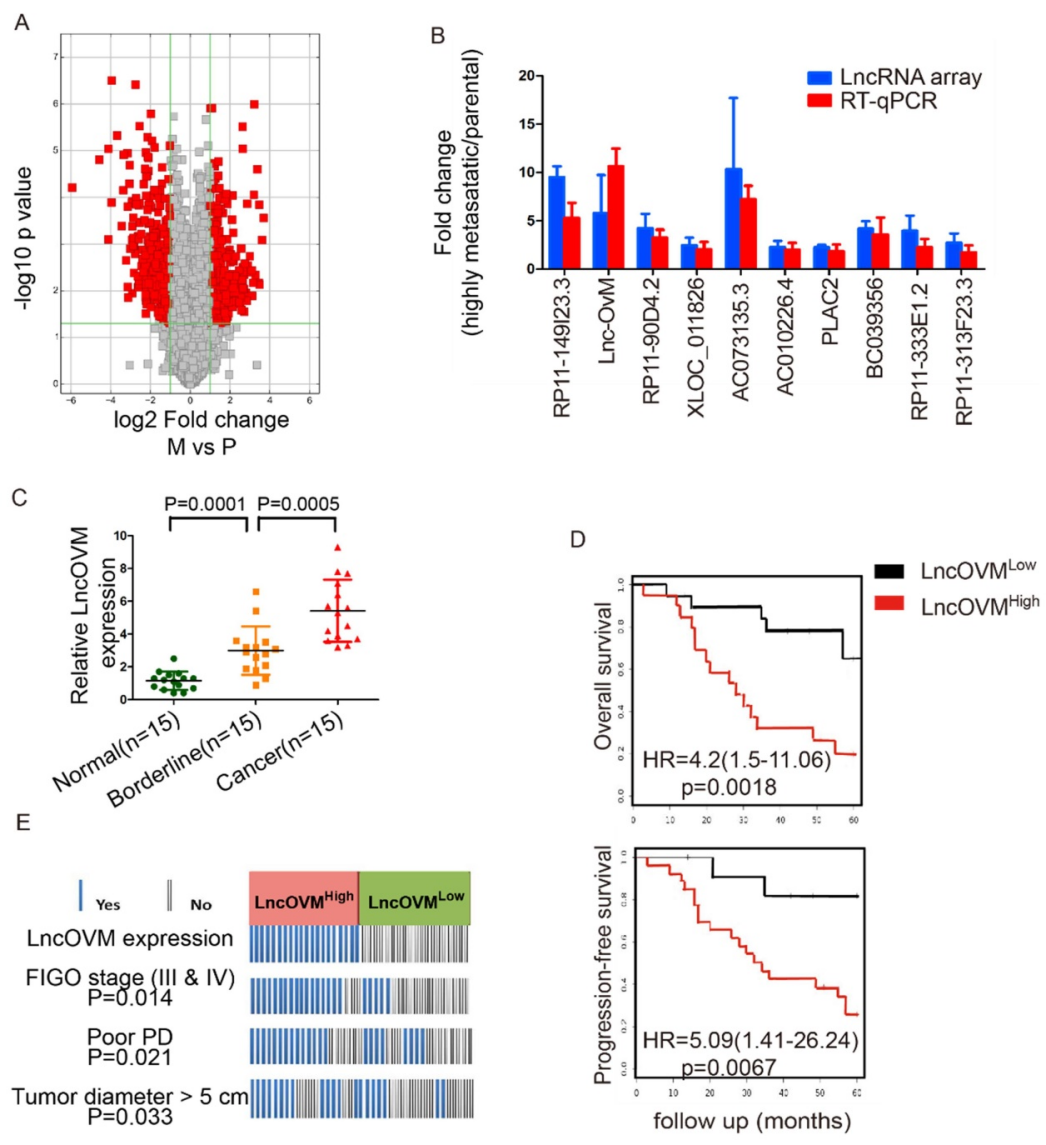
** LncOVM correlates with human metastatic ovarian cancer progression and poor prognosis.** A. The volcano plot visualization of the expression of LncRNA between human metastatic ovarian cancer tissue and parental tissues. The red dots represent up-regulated (right) and down-regulated (left) LncRNAs with statistical significance. B. LncRNA array and RT-qPCR data displaying the top 10 up-regulated LncRNAs involved in ovarian cancer progression. C. Relative expression of LncOVM in normal tissues (n=15), borderline tissue (n=15) and tumor tissues (n=15) from ovarian cancer patients detected by RT-qPCR. D. Kaplan-Meier survival curve of overall survival and progression-free survival in patients (n=20, each group) with ovarian cancer. Patients grouped by median of LncOVM expression level. E. Analysis of ovarian cancer patients with FIGO stage, progressive disease (PD) and diameters. Patients were stratified into high expression and low expression group by median expression (n=20, each group).

To explore important LncRNAs which may involve in ovarian cancer metastasis, we analysed the LncRNA profiles in highly metastatic ovarian cancer tissues and their parental tissues by microarray. After conducting volcano plot analysis (Fig. 1A), we validated top 10 up-regulated LncRNAs (RP11-149I23.3, LncOVM, RP11-90D4.2, XLOC_011826, AC073135.3, AC010226.4, PLAC2, BC039356, RP11-333E1.2 and RP11-313F23.3) by RT-qPCR. Compared with the parental tissues, the increased levels of LncOVM was the most significant in the high metastatic tissues (Fig. 1B).

To further investigate the clinical importance of LncOVM in ovarian cancer patients, we collected adjacent normal, borderline and cancer tissues from 15 ovarian cancer patients according to clinical pathology judgment. We then examined the expression levels of LncOVM by RT-qPCR in these tissues. Obviously, LncOVM was significantly higher in the borderline tissues than that in the normal tissues in the ovarian cancer patients (*p=*0.0001). LncOVM also dramatically increased in the cancer tissues as compared with the borderline tissues (*p=*0.0005, Fig. 1C), positively correlated with the ovarian cancer progression. We postulated that the elevation of LncOVM in the borderline tissues may indicate its important role in the tumor microenvironment that is related to ovarian cancer metastasis. We further analysed information from additional 40 ovarian cancer patients. We divided them into LncOVM^high^ (n=20) and LncOVM^low^ (n=20) groups. The LncOVM^high^ group showed poor survival rate as compared with the LncOVM^Low^ group in Kaplan-Meier overall survival analysis [*p*=0.0018, HR=4.2 (1.5-11.06), Fig. 1D]. Similarly, the patients with the low LncOVM level showed better progression-free survival rate [*p=*0.0067, HR=5.09 (1.41-26.24), FIG. 1D]. According to clinical parameters, the ovarian cancer patients with the high level of LncOVM were associated with a worse clinical outcome (Fig. 1E). Most patients in the LncOVM^high^ group were in advanced stages (FIGO stage III & IV), implying severe metastasis or extension of tumor (*p*=0.0014)^36^. Moreover, the patients with the high LncOVM level were also correlated to poor prognosis (PD, *p*=0.021) and larger tumor size (tumor diameter > 5 cm, *p*=0.033, Fig. 1E).

### LncOVM promotes ovarian cancer cell growth and metastasis

**Figure 2:**
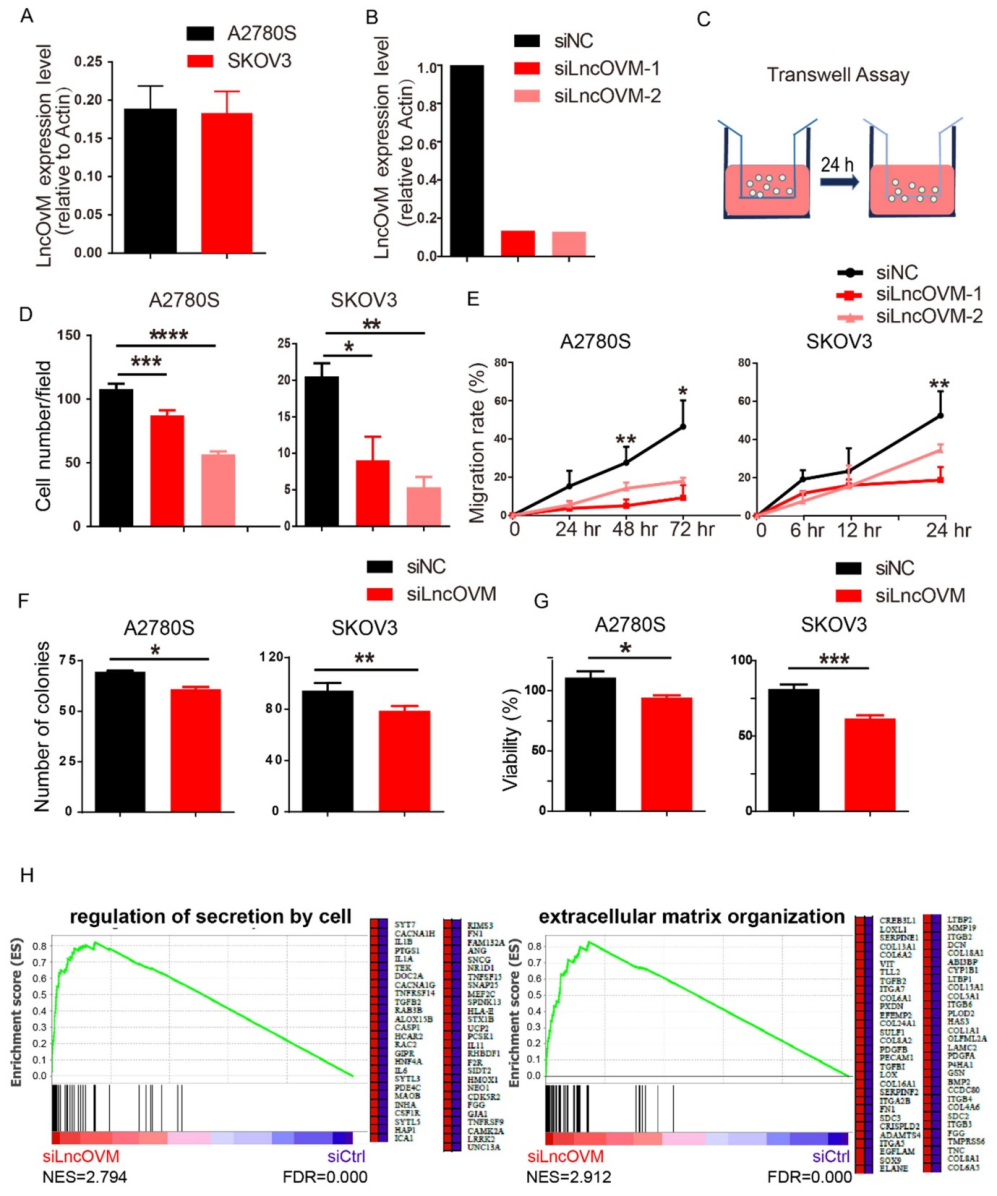
** LncOVM promotes ovarian cancer proliferation and metastasis *in vitro*.** A. Relative LncOVM expression levels in human epithelial ovarian cancer cells A2780s and SKOV3. B. Relative LncOVM expression levels in A2780S cells with the siLncRNAs transfection (siLncRNA-1/2) or with the negative control transfection (siNC). C. A schematic diagram of transwell assay. D. Statistic plot of transwell assays in A2780S and SKOV3 cells with siLncRNA or siNC. E. Wound healing analysis of A2780s and SKOV3 cells. F. Colony formation of A2780s and SKOV3. G. Cell viability of A2780s and SKOV3 measured by MTT assay. H. Gene Set Enrichment Analysis of RNA-seq data. *, *p*<0.05; **, *p*<0.01; ***, *p*<0.001; ****, *p*<0.0001.

We next asked whether LncOVM plays a role in promoting ovarian cancer metastasis. We knocked down LncOVM in invasive human ovarian cancer A2780s and SKOV3 cells, which had similar LncOVM levels to those ovarian cancer tissues (Fig. 2A & 2B). Knockdown of LncOVM significantly reduced the invasiveness of both A2780s and SKOV3 cells (Fig. 2C & 2D). The migration rates of LncOVM knockdown also significantly decreased as compared with the control cells (Fig. 2E). In addition, LncOVM depletion inhibited the proliferation of A2780s and SKOV3 cells as compared with the control cells through colony formation and MTT assays (Fig. 2F & 2G). Furthermore, we applied transcriptomic assays by RNA sequencing (RNA-Seq). We revealed the differential gene expression in A2780s cells between the LncOVM-depleted and control groups (Fig. S2A & S2B). After we conducted function annotation 37, 38, we observed a significant correlation of dysregulated genes with several pathways in metastasis and proliferation, such as cell junction, adhesion, division and death (Fig. S2C). Through Gene Set Enrichment Analysis (GSEA), we showed an obviously upregulated cytokine secretion by cell and extracellular matrix organization upon LncOVM depletion (Fig. 2H). Together, LncOVM has a great effect on promoting the proliferation and metastasis of ovarian cancer cells.

### LncOVM forms a complex with protein PPIP5K2

**Figure 3:**
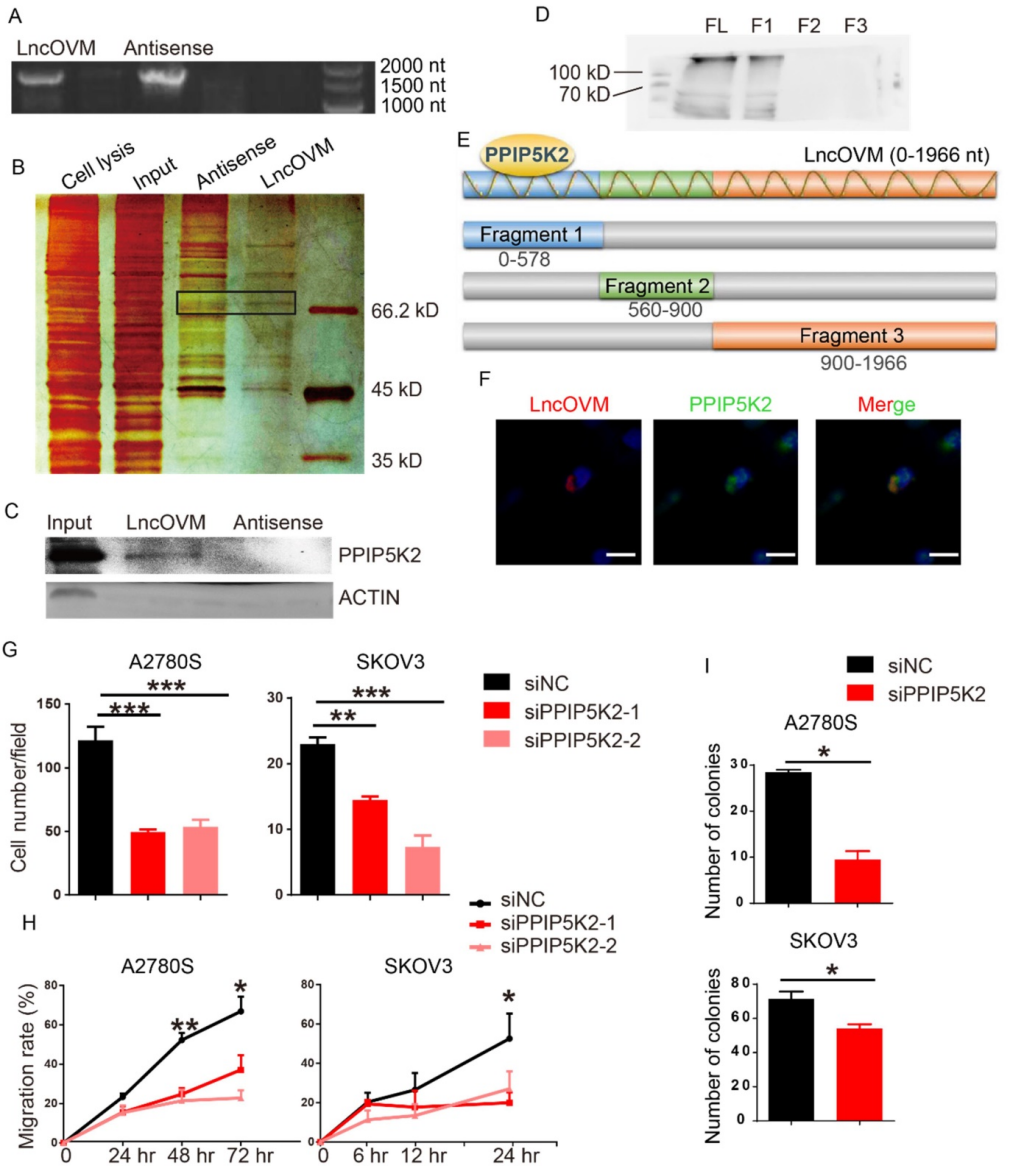
** LncOVM forms complexes with PPIP5K2 to promote ovarian cancer progression.** A. Agarose electrophoresis of LncOVM (full length, 1966 nt) and anti-sense RNA of LncOVM as a negatives control. B. The SDS-PAGE showing proteins bound to sense or antisense LncOVM. The highlighted region was submitted for mass spectrometry, and the protein PPIP5K2 was identified. C. Western blot analysis of proteins in sense and antisense LncOVM RNA pull-down solution from A2780S cell lysis, detecting by the indicated antibodies. D-E. Streptavidin RNA pull-down Assay by 3 RNA fragments and full length LncOVM as part E, a schematic diagram of RNAs fragments of LncOVM, followed by IB detection using the anti-PPIP5K2 antibody. F. Co-localization of PPIP5K2 and LncOVM detected by Immunofluorescence image in A2780S cells. LncOVM was hybridized with oligonucleotide probes with Cy3 fluorophore. G. Statistic plot of transwell assays in A2780S and SKOV3 cells with siPPIP5K2 or siNC. H. Wound healing analysis of A2780s and SKOV3 cells. I. Colony formation of A2780s and SKOV3 cells.

To understand the mechanism by which LncOVM promotes ovarian cancer progression, we sought to identify LncOVM interacting proteins. We employed a RNA pull-down assay with the full length LncOVM (1966 nt) and its antisense as control, followed by sliver staining and mass spectrometry (MS) (Fig. 3A & 3B). We revealed 134 interacting proteins bound to LncOVM, and 106 proteins bound to the antisense control (Fig. S3A). Using an *in vitro* RNA pull-down assay followed by Western blot assay, we further confirmed that PPIP5K2 is the LncOVM interacting protein, because it was only pulled down by LncOVM but not by its antisense control (Fig. 3C). To dissect the interacting region of PPIP5K2, we synthesized 3 overlapped fragments of LncOVM (Fig.3E) and repeated the RNA pull-down assays. Our results showed that the 5'-region (0-578 nt) interacted with PPIP5K2 (Fig. 3D). Furthermore, we further confirmed their intracellular interaction and colocalization by immunofluorescent assay. Obviously, LncOVM mainly co-localized with PPIP5K2 in 293T and A2780s cells although it speedily distributed in cytoplasm (Fig. 3F & S3B).

### PPIP5K2 promotes ovarian cancer progression

Previous studies showed that PPIP5K2 plays a role in many biological processes, such as apoptosis, vesicle trafficking, and cytoskeletal dynamics 23, 25, 26. To dissect its potential functions on the ovarian cancer progression, we knocked down PPIP5K2 to explore its effects on the metastatic ability of ovarian cancer cells (Fig. S4A). Surprisingly, both the invasion and migration dramatically decreased in cells with PPIP5K2 knockdown in transwell invasion assay (Fig. 3G) and wound-healing assay (Fig. 3H), respectively. To confirm the pro-tumor role of PPIP5K2 collaborating with the LncOVM, we generated a PPIP5K2-overexpressing A2780s cell line (Fig. S4B). Overexpression of PPIP5K2 with LncOVM-knockdown rescued their invasion capability (Fig. S4C). Besides, the knockdown of PPIP5K2 also reduced the proliferative ability of A2780s and SKOV3 cells (Fig. 3I). These data indicated that the PPIP5K2 promoted ovarian cancer progression collaborating with the LncOVM.

### LncOVM deficiency induces PPIP5K2 ubiquitination and degradation

**Figure 4:**
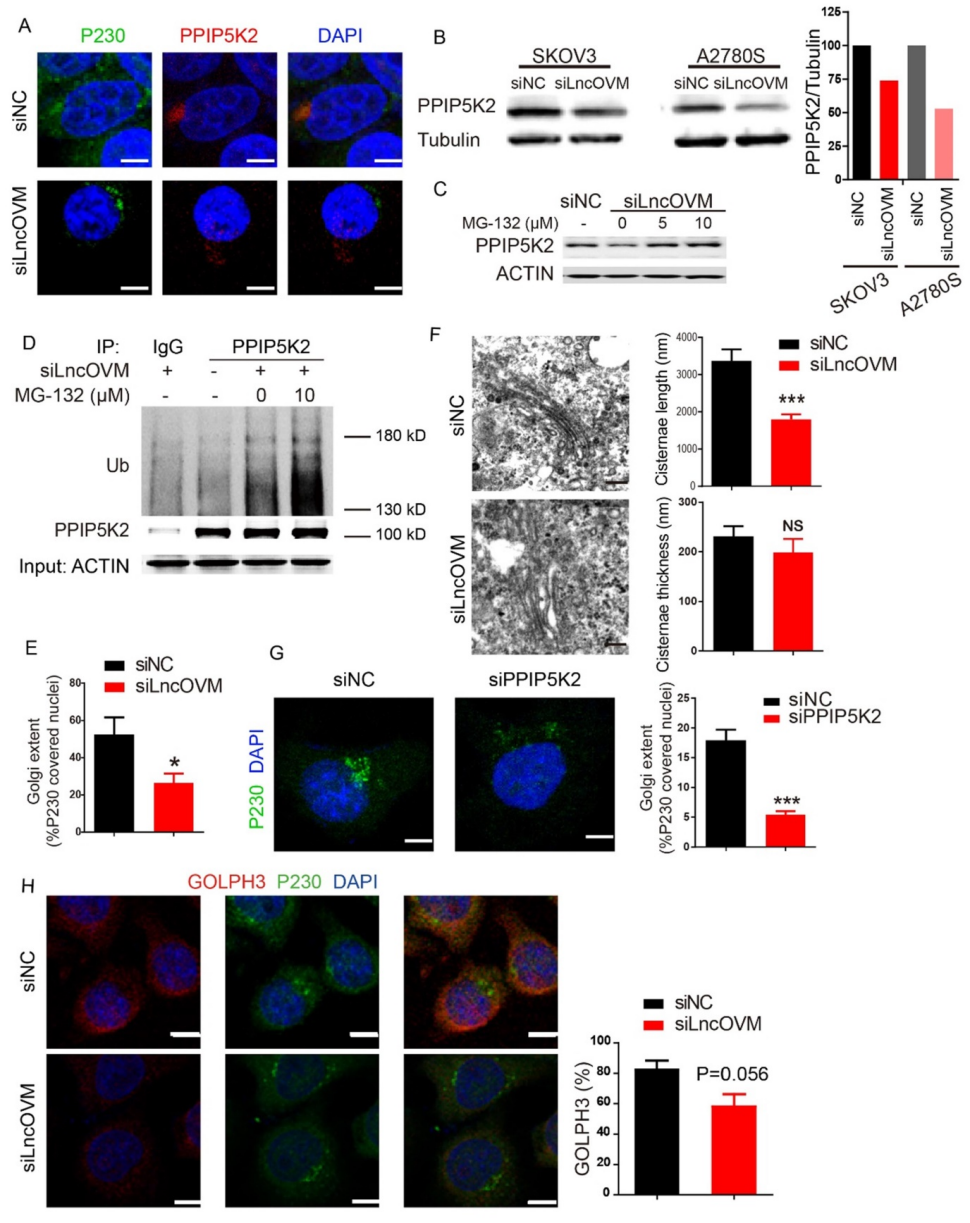
** LncOVM-PPIP5K2 complex protects PPIP5K2 from the ubiquitination and promotes Golgi extension.** A. Immunofluorescence analysis of PPIP5K2 and Golgi marker P230 in A2780S cells with siNC or siLncOVM. B. Western blot analysis of PPIP5K2 protein expression in A2780s and SKOV3 cells with siNC or siLncOVM. Data quantification showed on the right. C. Western blot analysis of PPIP5K2 protein expression in A2780s cells with MG-132 treatment (5 μM or 10 μM for 24 hours).D. Immunoprecipitation (IP) assays using the indicated antibodies in A2780s cells. IP with IgG as control and PPIP5K2, respectively. Size markers (in kDa) are depicted on immunoblot panels. E. Quantification for the Golgi extent in part A. The Golgi extent was quantified as the fraction of the nucleus circumference that was covered by P230-positive Golgi signal by ImageJ. F. Transmission electron microscope (TEM) images of Golgi structures in A2780S cells with siLncOVM or siNC. Quantification for Golgi cisternae length and thickness on the right**.** G. Immunofluorescence image with signal quantification for endogenous GOLPH3 and P230 in A2780S cells with siLncOVM or siNC. H. Immunofluorescence image for P230 and DAPI in A2780S cells with siPPIP5K2 or siNC. The Golgi extent was quantified on the right as the fraction of the nucleus circumference that was covered by P230-positive Golgi signal by ImageJ.

To further investigate the underline mechanism of cancer progression caused by LncOVM, we further explored whether and how LncOVM regulates PPIP5K2. In the LncOVM silenced cells, we observed that PPIP5K2 accumulation is significantly decreased in the Golgi complex, which was stained by a specific marker P230 (Fig. 4A). We also showed that depletion of LncOVM reduced the PPIP5K2 protein level by Western blot assay (Fig.4B). Consistent with the results from transcriptomic assays (Fig. S1B), knockdown of LncOVM had no effect on the PPIP5K2 mRNA level (Fig. S4D), indicating that LncOVM plays a crucial role in regulating PPIP5K2 degradation without affecting its transcription or mRNA stability. Importantly, upon treating cells with proteasome inhibitors MG-132, we observed that the loss of PPIP5K2 proteins mediated by LncOVM-depletion was completely restored (Fig. 4C). This revealed that the PPIP5K2 degradation caused by LncOVM-depletion was processed by proteasome. Results from co-immunoprecipitation (Co-IP) assay revealed that LncOVM knockdown caused a dramatically accumulation of ubiquitinated PPIP5K2 protein, and MG-132 treatment further increased its ubiquitination level of PPIP5K2 in A2780s cells (Fig. 4D). This further confirmed that the PPIP5K2 degradation mediated by LncOVM-depletion was through the ubiquitin-proteasome system. Our results indicated that LncOVM protected the PPIP5K2 stability from ubiquitin conjugation and proteasome degradation. Taken together, the physical interaction of LncOVM and PPIP5K2 suppressed the ubiquitination and subsequent proteasomal degradation of PPIP5K2 in ovarian cancer cells.

### LncOVM-PPIP5K2 complex has a fundamental role in the Golgi complex structure

PPIP5K2 is reported to localize in cytosol 39, but its precise subcellular localization has not yet been well investigated. We employed an immunofluorescence analysis to explore its subcellular localization. Results from our study showed that PPIP5K2 colocalized with in the Golgi marker P230 and accumulated in the Golgi complex in both A2780s and 293T cells (Fig. 4A & S3C).

Golgi complex, a vital subcellular organelle for processing protein secretion, consists cisternae stacked with a distinct polarity, trafficking cargo bud from cis face to trans face via vesicles in the forward vesicular-trafficking model. The trans face, which forms the trans Golgi network (TGN), is involved in packaging and delivering cargo to the plasma membrane 40, referring that the P230-marked TGN is associated with protein secretion 41. Previous studies reported that Golgi ultra-structure was alterable due to the dynamic of the actin cytoskeletal proteins, which interacted with the TGN member proteins. In actively secreting cells, the TGN structure stretched with more biogenesis of TGN-derived budding 42. The Golgi morphology change can be quantified by the cisternae length, cisternae thickness, and number of Golgi stacks 43. We employed both immunofluorescence and transmission electron microscopy (TEM) assays to investigate the effects of LncOVM depletion on the Golgi complex. Golgi condensation was observed when LncOVM was depleted in A2780s cells. LncOVM knockdown significantly reduced trans-Golgi P230 abundance (Fig. 4A & 4E), resulted in an intriguingly increased cisternae length but no obvious effect on the cisternae thickness, number of Golgi stacks or the number of Golgi-associated vesicles (Fig. 4F). Similar to LncOVM depletion, PPIP5K2 knockdown also inhibited the trans-Golgi P230 abundance (Fig. 4G). In the LncOVM or PPIP5K2 depleted cells, the Golgi complex presented more dispersed structure as compared with the normal cells. GOLPH3 is an important Golgi protein for promoting vesicle release. The increased GOLPH3 abundance in the TGN increases Golgi extension and vesicular release 44. Consistently, we observed that LncOVM knockdown significantly decreased the GOLPH3 level after we quantified GOLPH3 immunofluorescence image (Fig. 4H).

### PPIP5K2 regulates complement protein secretion

We further asked if any secretion proteins are impacted by PPIP5K2 and play crucial role in ovarian cancer progression. We collected the conditioned media from the PPIP5K2-depleted A2780s cells and the normal A2780s cells, then performed an iTRAQ secretomic analysis (Fig. S5A). We identified 630 proteins including 179 extracellular or secreted proteins according to UniPort annotated subcellular location. After bioinformatics analysis, 106 proteins showed significant change in cells with PPIP5K2 knockdown, and most of them were secretory proteins (Fig. S5B). We analyzed the biological function enrichment of those 106 proteins by Gene Ontology assays. We revealed that PPIP5K2 potentially activate immune response (Fig. S5C).

**Figure 5:**
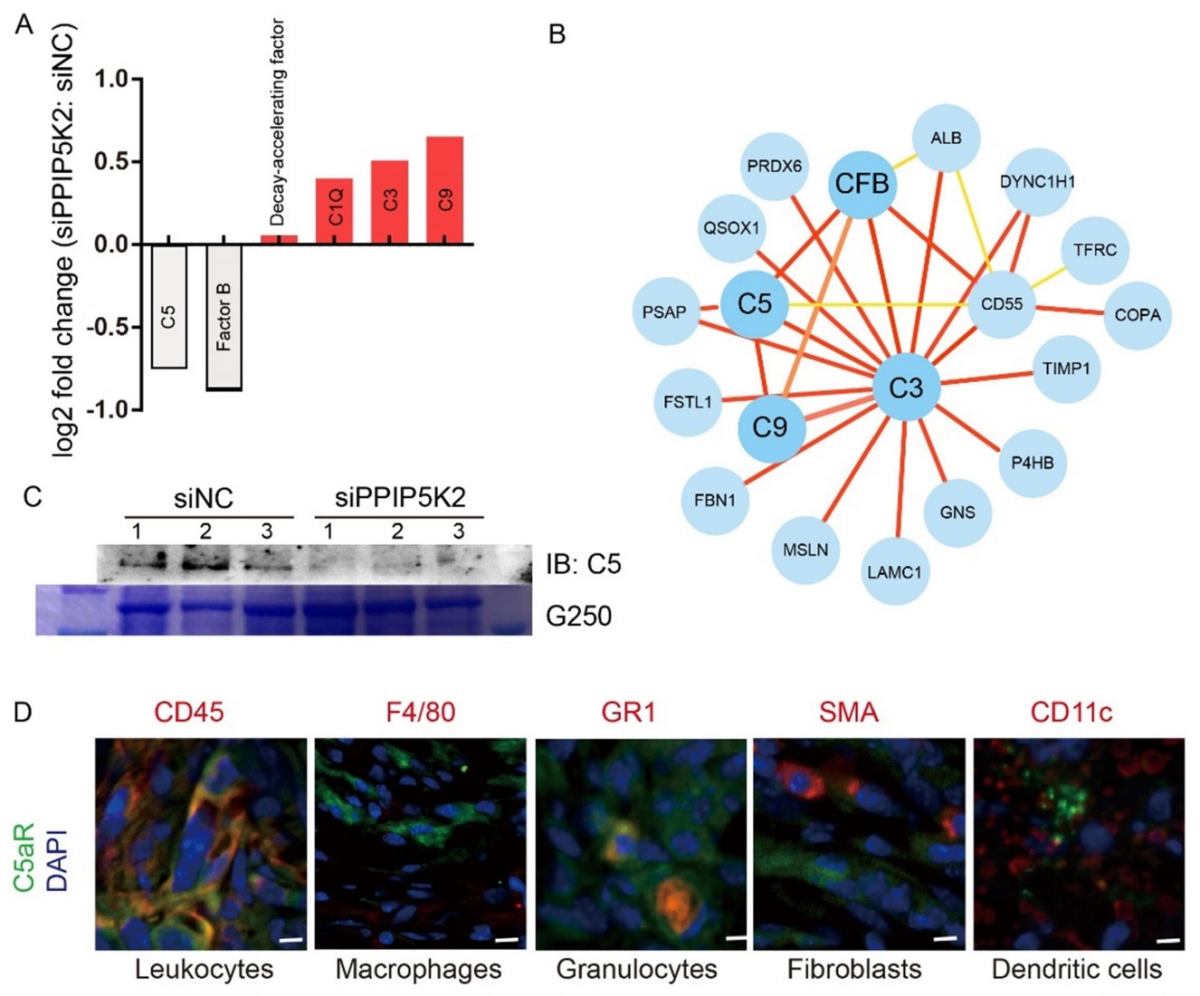
** PPIP5K2 facilitates malignant secretion including complement C5 which impacts MDSC recruitment.** A. Identified secretion proteins in complement system by iTRAQ with significant change. B. The protein-protein interaction network showing the complement factors connected proteins in iTRAQ data. C. Western Blot Verification of complement C5 in 100-fold concentrated conditional medium from 48-hour starved A2780S cells with siPPIP5K2 or siNC (n=3). Coomassie blue staining G250 was referenced as a loading control. D. Co-IF of C5aR1 (green) with indicated lineage markers for immune cells in ID8 tumor tissues from C57BL/6 mice.

We noticed that the abundance of complement proteins, closely associated with immune response 45, was markedly changed upon PPIP5K2 depletion (Fig. 5A). A network analysis by using STRING program^46^ (https://string-db.org/) showed that several complement proteins, including complement C3, C5, C9, complement factor B (CFB) and complement decay-accelerating factor (CD55) (Fig. 5B), were found to interact with those proteins involved in cell proliferation and migration (e.g., TIMP1 47, 48, P4HB 49, FBN1 50, 51, QSOX1 52, etc.). The results from our study raised a possibility that PPIP5K2 depletion dysregulates complement system that contributes to cell proliferation and migration.

### Complement C5 impacts MDSC recruitment

Complement system is not only a crucial component of innate immunity but also involved in the adaptive immune responses and inflammatory responses governing in tumorigenesis and cancer progression. It has been reported that complement system is activated and complement proteins (e.g., C5a) are up-regulated in various malignant tumors as well as cancer cell lines 53. C5, a key protein in the downstream of complement activation pathway, is cleaved to C5a and C5b. C5a is a potent anaphylatoxin for recruiting immune cells (i.e., neutrophils) to inflammation, tissue damage or tumor areas. C5a and its receptor C5aR are critical mediators in cancer immune response, a critical process for the development of pro-tumor microenvironment 8.

Consistent with complement C5 downregulation in the cells with PPIP5K2 knockdown in iTRAQ analysis, PPIP5K2 depletion significantly reduced C5 level in a 100-fold concentrated condition media of A2780s (Fig. 5C). More importantly, there was a positive correlation between the levels of PPIP5K2 and C5 *in vivo*. ID8 is an ovarian surface epithelium cell line from C57BL/6 mouse, which perfectly resembles human epithelial ovarian cancer in physiology and biology 54. PPIP5K2-depleted ID8 cells were subcutaneously inoculated in C57BL/6 mice to build an immunocompetent animal model. In this study, PPIP5K2 knockdown reduced tumor volume and weight, whereas intraperitoneally injected protein C5 (0.025 mg/kg, every second day) in PPIP5K2-depletion tumor led to an increase of tumor size as compared with the normal saline (NS) control, demonstrating that C5 promotes tumor progression as a downstream target of PPIP5K2 (Fig. S5D-F).

It has been reported that TME was dominated by tumor-induced interactions and enrichment of MDSCs (CD11b^+^, Gr-1^+^) 55. We further ask whether the secreted complement C5 affect immune cell infiltration in TME. Murine tumor tissues with TME were obtained from subcutaneously inoculated ID8 cell lines in C57BL/6 mice. The infiltration of C5aR^+^ immune cells in TME were identified by co-immunofluorescent staining. In our study, we identified C5aR^+^ cells including leukocytes (CD45^+^) and granulocytes (Gr1^+^, refers to Ly6C^+^ and Ly6G^+^), whereas C5aR was not detected on macrophages (F4/80^+^), fibroblasts (SMA^+^) and dendritic cells (CD11c^+^) (Fig. 5D), consistent with our results from flow cytometry (Fig. S1B & S1C), indicating the infiltrated granulocytes (Gr-1^+^) played an important role in our ovarian cancer tissues.

### Targeting LncOVM-PPIP5K2-Complement C5 axis suppresses MDSC infiltration in TME against cancer metastasis and tumorigenesis

**Figure 6:**
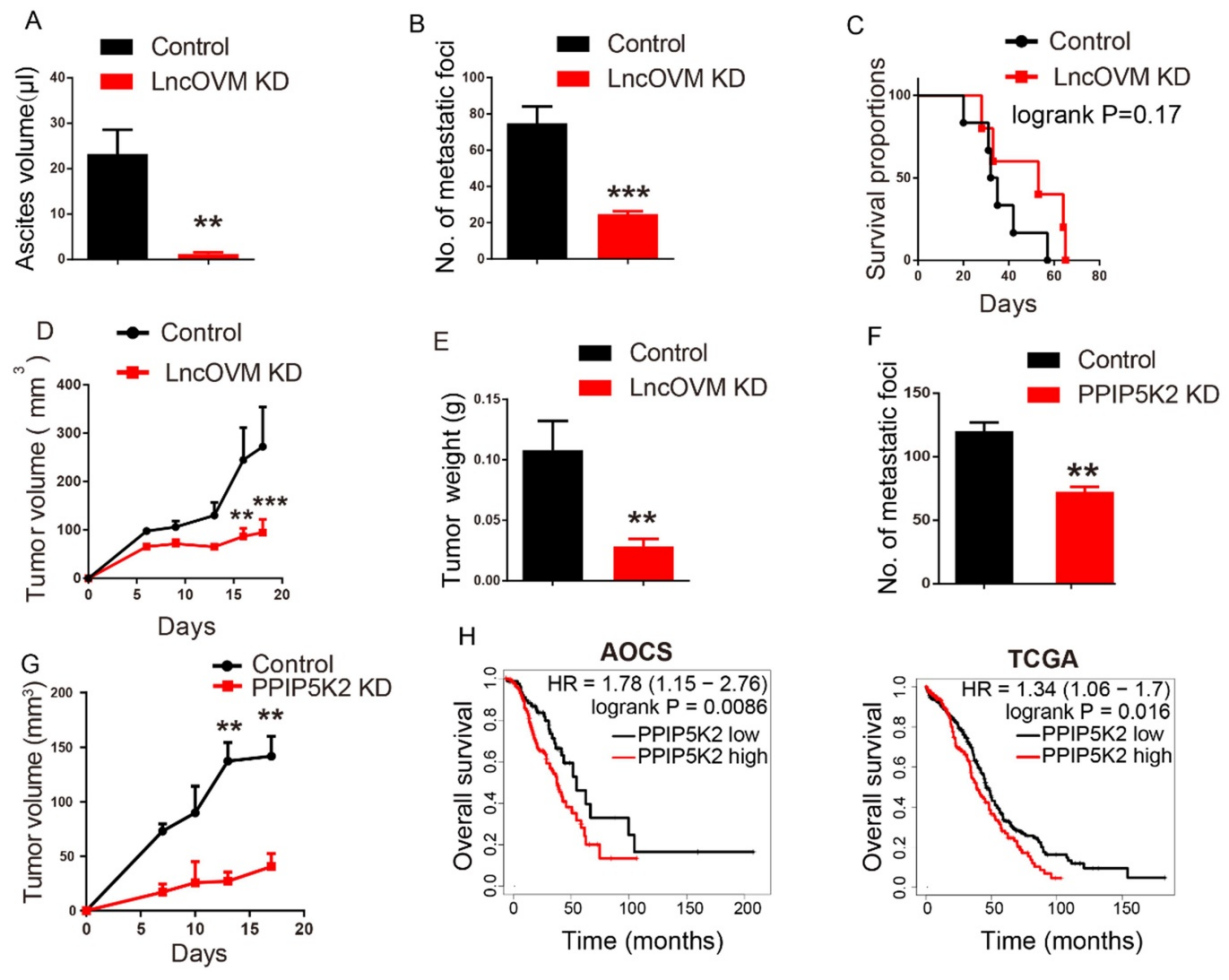
** Disrupting LncOVM-PPIP5K2 complex suppresses cancer metastasis and tumorigenesis.** A-B. Statistic plot of the ascites volumes collected from the abdominal cavities and number of metastatic nodules in the abdominal cavities of an intraperitoneally inoculation model. A2780S control cells (5x10^6^) and LncOVM knock-down (KD) cells were injected i.p. into 6-week balb/c nude mice (n=5). C. Kaplan-Meier survival curve of balb/c nude mice inoculated i.p. with A2780S control cells (5x10^6^) and LncOVM KD cells (n=6). D. Tumor growth curves in mice. A2780S control cells (1x10^7^) and LncOVM KD cells were inoculated s.c. into 6-week balb/c nude mice (n=5). E. Tumor weight of A2780S control and LncOVM KD mouse tumor tissues. Data were measured after dissection. F. Statistic plot of number of metastatic nodules in the abdominal cavities of an intraperitoneally inoculation model. A2780S control cells (5x10^6^) and PPIP5K2 KD cells were injected i.p. into 6-week balb/c nude mice (n=5). G. Tumor growth curves of mice. A2780S control cells (1x10^7^) and PPIP5K2 KD cells were inoculated s.c. into 6-week balb/c nude mice (n=5). H. Kaplan-Meier analysis of AOCS (n = 285) and TCGA (n = 565) patients with ovarian carcinoma showing a significant correlation between PPIP5K2 protein expression and overall survival.

The results from above studies reveal that LncOVM played crucial roles in multiple steps to trigger metastasis and TME remodelling. In ovary cancer patients, it is found that extensive dissemination and metastasis in the abdominal cavity often cause intestinal obstruction, diffuse peritoneal thickening, and severe ascites 56. To reveal the potential clinical value of anticancer, we first investigated the anticancer potential of LncOVM knockdown *in vivo*. Nude mice inoculated intraperitoneally with the LncOVM-depleted A2780s or control cells for 3 weeks. At sacrifice, it was found that LncOVM knockdown largely decreased ascites volume (*p* < 0.01) and metastatic nodules (*p* < 0.001) *in vivo* as compared with the control (Fig. 6A, 6B & S6A). LncOVM depletion increased the survival rate (Fig. 6C), and effectively inhibited tumor growth (*p* < 0.01, Fig.6D, 6E & S6B), as revealed by measuring tumor volume and weight. The metastatic markers also showed a significant change. A significant increase of E-cadherin and an obvious decrease of vimentin were observed by IHC staining in the LncOVM-depletion subcutaneous xenograft tumor tissues (Fig. S6C).

Next, we investigated the anticancer potential by knockdown of PPIP5K2 *in vivo*. We intraperitoneally inoculated PPIP5K2-depleted A2780s or control cells into nude mice. Compared with the control, PPIP5K2 depletion resulted in a significant decrease of metastatic nodules (Fig. S6D and 6F, *p* < 0.01). In addition, we built up a subcutaneous xenograft tumor model by using PPIP5K2-depleted cells. Again, we observed a significant decrease of tumor volumes (Fig. S6E and 6G) with obvious E-cadherin increase and vimentin decrease when PPIP5K2 was depleted (Fig. S6F). More importantly, the high level of PPIP5K2 expression was well corrected with poor prognosis of ovarian cancer patients accordingly, for instance, the overall survival that was revealed by Kaplan-Meier analysis on AOCS and TCGA databases (Fig. 6H).

**Figure 7:**
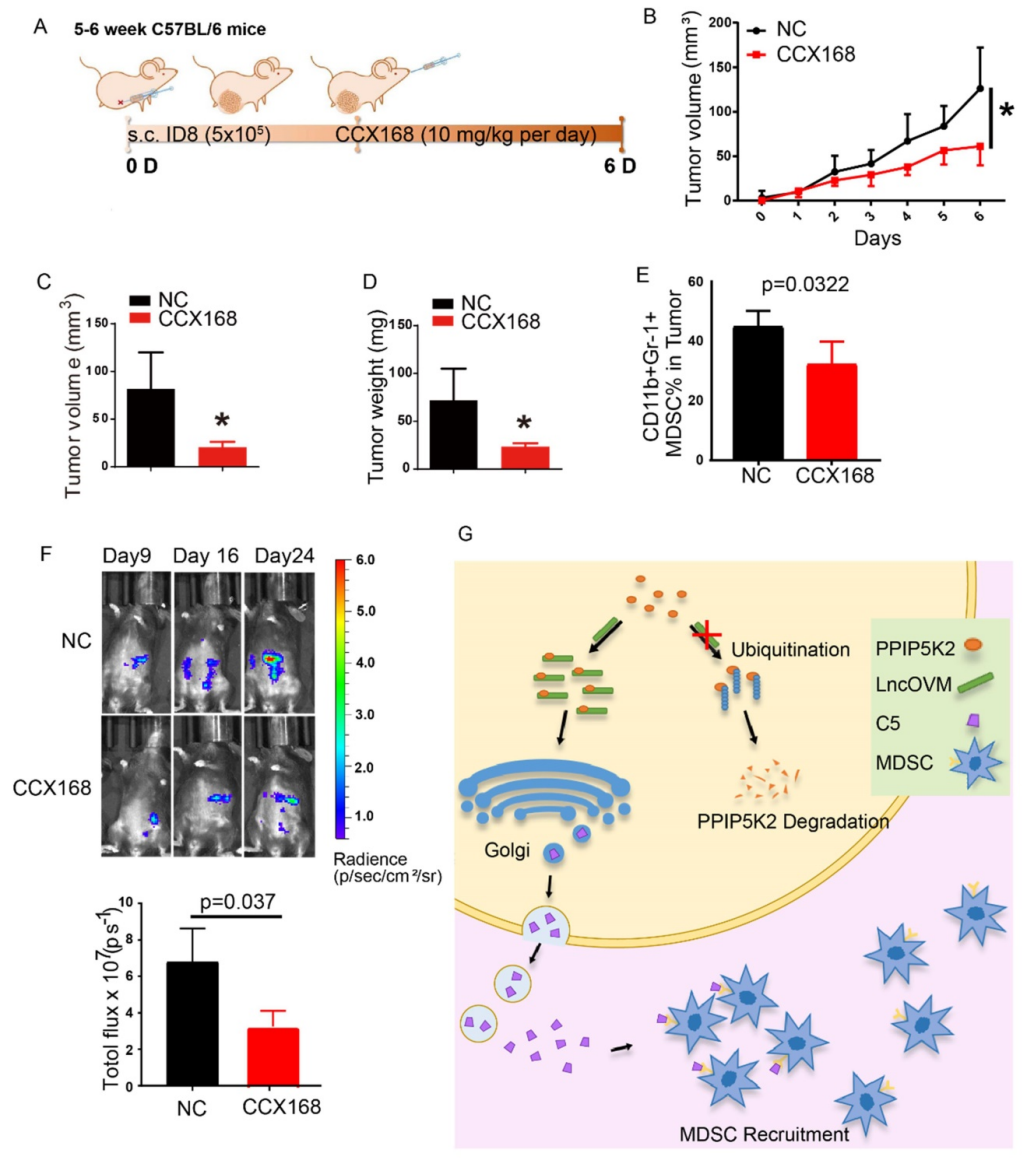
** The C5a receptor inhibitor suppresses MDSC infiltration in TME against cancer metastasis and tumorigenesis.** A. Schematic diagram of subcutaneous ID8-luciferase tumors in C57BL/6 mice with CCX168 treatment. The ID8-luciferase cells (5x10^5^) were inoculated into 6-week C57BL/6 mice subcutaneously. After inoculation, the CCX168 (10 mg/kg) or normal saline as control was given by oral gavage every day. B-D. Tumor growth curves, post-dissection measured tumor weights, and volumes of C57BL/6 mice treated with CCX168 or normal saline as control. ID8-luciferase cells (5x10^6^) were inoculated s.c. into 6-week C57BL/6 mice (n=6 per group). The C57BL/6 mice were treated with CCX168 (C5aR inhibitor, 10 mg/kg) or by oral gavage every day. E. The flow cytometry analysis showed fraction change of MDSCs (CD11b+, Gr-1+) in ID8 mice tumor microenvironment. F. Bioluminescence imaging of intraperitoneal ID8-luciferase tumors in C57BL/6 mice with CCX168 treatment. The ID8-luciferase cells (5x10^5^) were inoculated i.p. into 6-week C57BL/6 mice. After 9-day inoculation, the CCX168 (10 mg/kg) or normal saline as control was given by oral gavage every day. Statistic plot of total flux in bioluminescence imaging. G. The graphic illustration of LncOVM-mediated PPIP5K2 regulating complement C5 secretion through the Golgi complex and MDSC recruitment in tumor microenvironment.

At last, we investigated the potential application of the inhibitor of C5a receptor and C5a antibody for ovarian cancer treatment. CCX168 (avacopan) is a safe and clinical effective inhibitor of the orally-administered for C5a receptor 57 with a completed clinical trial 58. We established a subcutaneous ID8-luciferase cell inoculation tumor model (Fig. 7A). CCX168 in 0.1 ml PBS (10 mg/kg per day) or NS control was delivered by oral gavage to mice model*.* The block of C5aR revealed the inhibition of tumor progression as measuring the tumor volume and weight (Fig. 7B-D). Besides, the C5aR inhibitor CCX168 also down-regulated MDSC proportion (*p*=0.0322) in tumor microenvironment (Fig. 7E). After 9 days, the mice bearing tumor were separated into two groups evenly, and CCX168 (10 mg/kg per day) was administered by oral. Bioluminescence imaging was performed on day 9, day 16 and day 24 and the total flux showed a significant suppression in CCX168 group (Fig. 7F). Our data suggested that the C5aR inhibitor CCX168 suppressed the tumorigenesis and metastasis.

Besides, a C5aR antibody (α-C5aR, HM1076, HycultBiotech) was also subcutaneously injected into ID8 tumor surrounding surface (0.6 mg/kg, every second day) in C57BL/6 mice (Fig. S7A). Inhibition of C5aR with the C5aR antibody led to a slightly inhibition of tumor growth (Fig. S7B) and a decrease of MDSC proportion (*p* < 0.01) in tumor microenvironment (Fig. S7C & S7D). Both results showed that PPIP5K2-regulated complement C5 recruited MDSC to remodel the tumor microenvironment. Furthermore, the inhibition of C5a receptor effectively suppressed the ovarian cancer progression.

## Discussion

PPIP5K2, a bifunctional inositol kinase, has never been known its function in cancer, although it is reported to regulate various biological processes such as apoptosis, vesicle trafficking, cytoskeletal dynamics, and exocytosis 23-25. In this study, we explored the functions and mechanism of the LncOVM-PPIP5K2 complex in regulating complement secretion. More importantly, we showed that both C5aR inhibitor CCX168 and complement C5 antibody inhibited metastasis and extended tumor-loaded mice survival by inhibiting MDSC infiltration in TME.

In our *in vivo* practice, we observed MDSC infiltration in the TME of ovarian cancer patients (Fig. S1A & S1B). To understand the underline mechanism and develop optional measures for preventing and treating ovarian cancer, we employed LncRNA microarray study to discover metastasis-associated LncRNAs. We revealed that LncOVM was correlated with metastatic progression in ovarian cancer. LncOVM formed a complex with protein PPIP5K2 and remodeled the structure of Golgi complex, leading to the enhanced complement C5 secretion and MDSC infiltration in TME (Fig. 7G), which promoted cancer metastasis.

Although PPIP5K2 is known to be associated with the survival risk of colorectal cancer 27, 59, 60; to our knowledge, its function has not been reported yet in ovarian cancer. We showed that PPIP5K2 was associated with the progression of ovarian cancer. Furthermore, we revealed that the interaction of LncOVM and PPIP5K2 protected the later from ubiquitination-mediated degradation. The LncOVM-PPIP5K2 complex coordinated the structure of Golgi complex. The extent and length of Golgi complex were reduced in ovarian cancer cells with depletion of LncOVM or PPIP5K2, which were with lower metastasis level compared with the wild type ovary cancer cells. It was reported that the extended Golgi complex could be a morphological feature of highly metastatic cells in breast and colon cancers 43, 44, 61. Our study on ovary cancer further validated and extended these findings, suggesting a common biological process for cancer metastasis. In addition, our proteomic analysis revealed that PPIP5K2 depletion affected the secretion of various proteins, many of which have been implicated in the immune response, including the neutrophil activation and neutrophil mediated immunity. The proteins in complement system display a dysregulation, and the activation of complement system in the tumor microenvironment promotes tumorigenesis 10, 62. In our study, the complement C5 secretion was found to be impaired in PPIP5K2 depleted cells. Moreover, we demonstrated that complement C5 was a big player in recruiting MDSCs into TME, leading to ovarian cancer progression. Our findings also support the findings obtained in other cancers 11, 32, 63. More importantly, results from our studies revealed that C5aR inhibitor CCX168, a safe and efficient treatment of antineutrophil cytoplasmic antibody-associated vasculitis, displayed an ideal potential for ovarian cancer therapy.

In summary, we revealed a novel mechanism of MDSC infiltration in TME through LncOVM-PPIP5K2-complement C5a axis for cancer metastasis (Fig.7G). LncOVM may be used as an early diagnostic marker of ovarian cancer. Suppressing MSDC infiltration could serve as an option for treating ovarian cancer patients through disrupting the signaling of LncOVM-PPIP5K2-complement axis with C5aR inhibitor or C5 antibodies.

## Materials and Methods

### LncRNA microarray analysis

The Human LncRNA Microarray V3.0 (Arraystar Inc.) was performed by KangChen Bio-Tech (Shanghai, China). The array was scanned by the Agilent Scanner G2505B (Agilent Technologies) and the acquired array images were analyzed by Agilent Feature Extraction software (version 10.7.3.1; Agilent Technologies). Quantile normalization and subsequent data processing were performed using the Gene Spring GX v11.5.1 software package (Agilent Technologies). The accession number for the microarray data in Gene Expression Omnibus database (GEO): GSE82059.

### Real-Time PCR (RT-PCR)

Total RNA was extracted from cells using RNA Isolation kit (TIANGEN^®^, China) according to the manufacturer's instructions. Reverse transcription was performed using a PrimeScript RT reagent kit (TaKaRa, Japan), and qRT-PCR was performed with iTaq™ Universal SYBR^®^Green Supermix (Bio-Rad, USA) using a CFX96 Touch™ Real-Time PCR Detection System (Bio-Rad, USA). Data were analyzed using the 2-ΔΔCt method.

### Cell culture

The human ovarian cancer cells SKOV3 and A2780s, and a human embryonic kidney 293T cells were obtained from ATCC. The mouse ovarian cancer cell line ID8 was a gift from the State Key Laboratory of Biotherapy, Sichuan University. The cells were maintained in Dulbecco's Modified Eagle's Medium (DMEM, Gibco, USA) containing 10% FBS, 100 U/mL penicillin G, and 100 mg/mL streptomycin with 5% CO_2_ at 37°C.

### Animal experiments

All animal experiments were reviewed and approved by the Institutional Ethics Committee of Sichuan University. The female athymic BALB/c nude mice (5-6 weeks old, 16-20 g each) were used to establish the intraperitoneal xenograft tumor model of human ovarian cancer as described previously 20. The number of metastatic nodules was counted and ascites volumes were measured at sacrifice 20. For the subcutaneous tumor model, the 1x10^7^ A2780s cells were inoculated into the right flank of nude mice (female, 5-6 weeks old, 16-20 g each), and 1x10^7^ ID8 cells were inoculated into the right flank of C57BL/6 mice (female, 6-8 weeks old, 18-20 g each). For ID8-luciferase cell intraperitoneal and subcutaneous tumor model, 5x10^5^ and 5x10^6^ ID8-luciferase cells were inoculated into C57BL/6 mice, respectively.

### Clinical samples

A total of 45 ovarian tumor samples paired with adjacent tissues were obtained for patients in West China Second Hospital, Sichuan University (Sichuan, China). All these samples were obtained by experienced gynecological oncologist and examined by experienced pathologists who confirmed the diagnosis of disease samples. The fresh tissues were frozen in liquid nitrogen to protect the protein or RNA away from degradation. This study was approved by the Institutional Ethics Committee of Sichuan University. Informed consents were obtained from all patients prior to analysis.

### RNA sequencing

All RNA sequencing was performed at Novogene Co., Ltd. For sample treatment and collection, two groups of replicants were set up, which were A2780s cells transfected with two siRNAs, siNC and siLncOVM, separately. At least 1 μg RNA per sample was collected for RNA library preparation. RNA libraries were prepared using NEBNext Small RNA Library Prep Set for Illumina. High quality sequence data (Q30 ≥80%) were obtained by an Illumina HiSeq4000. The down-regulated group was defined as the ratio of siLncOVM/siNC ≤0.4, while the up-regulated group was defined as the ratio ≥5. DAVID analysis tools was used for functional annotation of interesting genes 37, 38, and then visual displayed by ggplot2 package.

### Cell transfection

According to the manufacturer's protocol, siRNAs or plasmids were transfected in cells using Lipofectamine 3000 (Invitrogen, USA). The plasmids for expressing the full-length, and fragments of LncOVM were purchased from TSINGKE Biologic Technology Co., Ltd; siRNAs were purchased from RiboBio Co., Ltd, Guangzhou, China. Plasmid for expressing sh-LncOVM, Lentiviral shPPIP5K2 and oe-PPIP5K2 were purchased from Shanghai Integrated Biotech Solutions Co., Ltd.

### Cell proliferation assay

Equal numbers of cells were seeded in 96-well plates and cultured for 24 or 48 hours. Cell viability was measured with MTT reagent 64. Cells were incubated with 5 mg/ml MTT for 4 hours at 37°C, and absorbance was measured at 590 nm.

### Colony formation assay

For the colony formation assay, cells were seeded into 6-well plates (100, 200 or 500 cells/well). The cells were cultured for 7 days in 37°C incubator. Subsequently, the colonies were fixed in 4% paraformaldehyde for 15 min at room temperature and stained with 5% crystal violet for 10 minutes at room temperature. Counts of the colony numbers were performed using ImageJ.

### Transwell assay

A2780s and SKOV3 cells (1x10^4^ cells/well) were harvested and seeded with serum-free DMEM into the Millicell Hanging Cell Culture (24 well, PET, 8um, Millipore) which hanged on the upper chambers of 24-well plates. The bottom chambers contained DMEM with 10% FBS. Cells were incubated for 24 hours at 37°C. Following incubation, the invaded cells attached to the lower surface of the membrane. The upper cells and medium were removed by swab, the invaded cells were fixed by 4% paraformaldehyde for 15 min at room temperature and stained with 5% crystal violet for 10 minutes at room temperature. The cells numbers were counted using ImageJ.

### Wound healing assay

Cells were seeded in 6-well plates at a similar density. After incubation for 24 h with blank medium, a 10-μl pipette tip was applied to generate a straight scratch, which simulated a wound. The cells were rinsed with medium twice to remove any floating cells and were then cultured in medium. Wound healing was observed at time points, and the scratch area was photographed. Triplicate wells were used for each condition, and each experiment was performed three times 65.

### Western blot assay

Intracellular proteins were extracted from cells by RIPA lysis buffer (Beyotime, P0013B, China) containing protease inhibitors at 4°C. Extracellular proteins were collected from conditional medium and concentrated 100-fold by centrifugation at 4000 rpm, 4°C with 30K ultrafiltration centrifugal devices (Thermo Scientific™ Pierce™). Each conditional medium sample was the serum-free DMEM-Flex media incubating with A2780s cells (3x10^8^) for 48 hours. Proteins were loaded to 10% SDS-PAGE gels and transferred onto nitrocellulose membranes. The membranes were hybridized with a primary antibody at 4°C overnight and incubated with a secondary antibody in 2h at room temperature. The expression of β-Tubulin was used as loading control.

The information of antibodies was listed as follow: PPIP5K2 (1:1000, Abcam, ab204374), β-Tubulin (1:1000, Proteintech, 11224-1-ap), C5 (1:500, Santa Cruz, SC-70476).

### RNA pull-down assay and mass spectrometry analysis

The biotin-labeled LncOVM and the antisense RNA were transcribed in vitro and purified with a Ribo^TM^ RNAmax-T7 biotin-labeled transcription kit (R11074, RiboBio Co., Ltd, Guangzhou, China.). RNA was refolded in structure buffer (10mM Tris, 0.1M KCl, 10 mM MgCl2) at 90°C for 2 min, on ice for 2 min and then stayed at room temperature for 30 min before pull-down. The cell lysates used for pull-down assay were prepared with Anti-RNase, Protease Inhibitor Cocktail, Phosphatase Inhibitor Cocktail in the lysis buffer^43^, and incubated with biotin-labeled RNAs (50 pmol) at 4°C overnight^66^. Dynabeads® MyOneTM Streptavidin C1 (Invitrogen, 65002) were prepared according to manufacturer's instructions. Beads were immediately added to the mixture and then incubated at 4°C overnight. The captured proteins were elated and analyzed by Mass Spectrometry 66.

### Co-Immunoprecipitation

Cell lysates were prepared and precleared for 1 h at 4°C with protein A/G-agarose (Sigma). Anti-PPIP5K2 and normal IgG as a negative control were incubated with cell lysates at 4°C overnight. The immunoprecipitation complex was pelleted by centrifuged at 1,000 x g at 4°C. After washing three times with lysis buffer, immune complexes were resolved by SDS-polyacrylamide gel electrophoresis (PAGE) and transferred to nitrocellulose membranes (Bio-Rad). A2780s cells treated with MG-132 (10 μM) for 24 hours at 37°C.

### Isobaric tags for relative and absolute quantitation (iTRAQ)

Secreted proteins in conditional media from control and PPIP5K2-depleted cells were analyzed by iTRAQ. A2780s cells (5 x10^7^) were maintained in serum-free DMEM-Flex media for 48 hours and the conditional media was collected. Proteins were identified using AB SCIEX TripleTOF™ 5600 plus in Wuhan GeneCreate Biological Engineering Co., Ltd., China.

### Immunohistochemistry (IHC)

Murine ID8 tumor tissues were fixed in 4% paraformaldehyde for 24 hours at room temperature. Immunohistochemistry for the target molecules was performed on paraffin sections using a primary antibody against E-cadherin (1:100, ZSGB-BIO, za-0565), Ki-67 (1:100, ZSGB-BIO, zm-0167) and Vimentin (1:100, ZSGB-BIO, za-0511).

### Transmission electron microscopy

Cells were fixed in 2.5% glutaraldehyde at room temperature for 30min, and embedded with the resin in Wuhan Servicebio Technology Co., Ltd. Images were acquired on an electron microscopy (TEM Hitachi H-7650). Quantification of the Golgi structures were performed using ImageJ.

### Immunofluorescence

A2780s and 293T cells were fixed in 4% paraformaldehyde for 30 minutes at room temperature. Murine ID8 tumor tissues were fixed in 4% paraformaldehyde for 24 hours at room temperature and were dehydrated in 30% sucrose for over 48 hours. The prepared tissues were store at -80°C for frozen sections. The tissues embedded in OCT at -25°C. 8.0 mm sections were air dried for 30 min. Sections and cells were permeabilized for 15 minutes in PBS with 0.5% Tritonx-100 and washed in PBST (PBS with 0.1% Tween 20). Blocking was carried out in 5% goat serum for 30 minutes at room temperature. Primary antibodies including: P230 (Biolegend, 611280, 1:100), Golph3 (Abcam, ab91492, 1:100), PPIP5K2 (Abcam, ab204374, 1:100), C5aR (Proteintech, 21316-1-AP), Ly-6G/Ly-6C (Gr-1) (Biolegend, 108401,1:100), CD45 (Biolegend, 103132, 1:100), CD11c (BD, 553801, 1:100), F4/80 (Biolegend, 123116, 1:100), SMA (ZSGB-BIO, zm-0003, 1:100) incubated overnight at 4°C. Primary antibodies were detected using the appropriate Alexa Fluor-labeled secondary antibodies (Invitrogen, 1:500). Slides were washed and incubated for 5 min with DAPI. Images were taken with an LSM710 Confocal Laser Scanning Microscope (Zeiss). Image analysis was performed using ImageJ software.

### Fluorescent in situ hybridzation (FISH)

PPIP5K2-overexpressing A2780s cells and 293T cells were briefly rinsed with PBS and then fixed with 4% paraformaldehyde for 10 min at room temperature. Cells were permeabilized in PBS containing 0.5% Triton X-100 for 5 min at 4°C, then washed with PBS for 5 min. 200 μL of Pre-hybridization Buffer was added at 37°C for 30 min. Hybridization was carried out with a FISH probe in a moist chamber at 37°C in the dark overnight using Ribo™ LncRNA FISH kit (C10910, RiboBio Co., Ltd, Guangzhou, China). The slides were washed three times with Wash Buffer I (4 × SSC with 0.1% Tween-20), once each with Wash Buffer II (2 × SSC), Wash Buffer III (1 × SSC) at 42°C in the dark for 5 min and once with PBS at room temperature. LncOVM-Cy3 FISH probes were designed and synthesized by RiboBio Co., Ltd. All images were obtained with an LSM710 Confocal Laser Scanning Microscope (Zeiss).

### Flow cytometry

Single-cell suspensions were prepared from ID8 tumor tissues from C57BL/6 mice. Tissue was manually minced using scissors, followed by a 60 min enzymatic digestion with 2.0 mg/ml collagenase A (Roche), and 50 U/ml DNase I (Roche) in serum-free DMEM at 37°C using continuous stirring conditions (Invitrogen). The tumor digests were passed through 70-mm nylon strainers (BD Biosciences). Subsequently, cells were incubated for 30 min with 100 ml of fluorophore-conjugated anti-mouse antibodies: C5aR (1:100, proteintech, 21316-1-AP), PE-Ly-6G/Ly-6C (GR1, 1:100, BD, 553128), FITC-CD11b M1/70 (1:100, BD, 553310). Cells were then washed once in PBS. Immediately after the wash, data acquisition on a BD Fortessa using FACS Diva software (BD Biosciences). Analysis was performed using FlowJo software program (Tree Star Inc).

### Statistical analysis

GraphPad Prism (Graph-Pad Software Inc.) was applied for data analysis for normal distribution and equal variance. The correlation study was performed using a linear regression analysis. Comparisons between two groups were performed by Student *t* test, and the differences among multiple groups were evaluated by one-way ANOVA. For incRNA array, the volcano plot was used to select the top 10 up-regulated LncRNAs involved in ovarian cancer progression. The threshold is fold change >1.3 or fold change <0.5 and *p* value < 0.05. The survival of different treatment groups was analyzed by Kaplan-Meier analysis. Kaplan-Meier survival analyses for disease outcomes in Australian Ovarian Cancer Study (AOCS) dataset (n =285), and The Cancer Genome Atlas (TCGA) dataset (n =565), were conducted using the online database (www.kmplot.com). Patients were stratified into "low" and "high" expression based on autoselect best cutoff in the database. The clinical stages of the patients in these two cohorts range from FIGO stage I to stage IV. P values were calculated with log-rank (Mantel-Cox) test. Patients were stratified into "low" and "high" expression based on medium. All data was considered significant when *p<0.05.

## Supplementary Material

Supplementary figures.

## Abbreviation

α-C5aR: C5aR antibody
AOCS: Australian Ovarian Cancer Study
C3: Complement component 3
C5: Complement component 5
C9: Complement component 9
C5aR: C5a receptor
CD55: Complement decay-accelerating factor
CFB: Complement factor B
FIGO: The International Federation of Gynecology and Obstetrics
FISH: Fluorescent in situ hybridization
GOLPH3: Golgi phosphoprotein 3
GSEA: Gene Set Enrichment Analysis
IB: Immunoblot
IF: Immunofluorescence
IHC: Immunohistochemistry
IP: Immunoprecipitation
iTRAQ: Isobaric tags for relative and absolute quantitation
KD: Knock down
LncRNA: Long noncoding RNAs
LncOVM: Metastasis-associated LncRNA in Ovarian Cancer
MDSC: Myeloid-derived suppressor cell
MS: Mass spectrometry
NC: Negative control
NS: Normal saline
PD: Progressive disease
PPIP5K2: Inositol hexakisphosphate and diphosphoinositol-pentakisphosphate kinase 2
TCGA: The Cancer Genome Atlas
TEM: Transmission electron microscopy
TGN: Trans Golgi network
TME: Tumor Microenvironment

## References

[1] Torre LA, Trabert B, DeSantis CE (2018). Ovarian cancer statistics, 2018. CA Cancer J Clin.

[2] Lheureux S, Gourley C, Vergote I, Oza AM (2019). Epithelial ovarian cancer. Lancet.

[3] Jayson GC, Kohn EC, Kitchener HC, Ledermann JA (2014). Ovarian cancer. Lancet.

[4] Schulz M, Salamero-Boix A, Niesel K (2019). Microenvironmental Regulation of Tumor Progression and Therapeutic Response in Brain Metastasis. Front Immunol.

[5] Jiang Y, Wang C, Zhou S (2020). Targeting tumor microenvironment in ovarian cancer: premise and promise. Biochim Biophys Acta (BBA)-Reviews Cancer.

[6] Scharping NE, Menk A V, Moreci RS (2016). The Tumor Microenvironment Represses T Cell Mitochondrial Biogenesis to Drive Intratumoral T Cell Metabolic Insufficiency and Dysfunction. Immunity.

[7] Arneth B (2019). Tumor microenvironment. Medicina.

[8] Medler TR, Murugan D, Horton W (2018). Complement C5a Fosters Squamous Carcinogenesis and Limits T Cell Response to Chemotherapy Article Complement C5a Fosters Squamous Carcinogenesis and Limits T Cell Response to Chemotherapy. Cancer cell.

[9] Afshar-Kharghan V (2017). The role of the complement system in cancer. J Clin Invest.

[10] Reis ES, Mastellos DC, Ricklin D (2018). Complement in cancer: untangling an intricate relationship. Nat Rev Immunol.

[11] Hsieh C-C, Chou H-S, Yang H-R (2013). The role of complement component 3 (C3) in differentiation of myeloid-derived suppressor cells. Blood.

[12] Peinado H, Zhang H, Matei IR (2017). Pre-metastatic niches: organ-specific homes for metastases. Nat Rev Cancer.

[13] Browning L, Patel MR, Horvath EB (2018). IL-6 and ovarian cancer: inflammatory cytokines in promotion of metastasis. Cancer Manag Res.

[14] Lokshin AE, Winans M, Landsittel D (2006). Circulating IL-8 and anti-IL-8 autoantibody in patients with ovarian cancer. Gynecol Oncol.

[15] Lane D, Matte I, Rancourt C, Piché A (2011). Prognostic significance of IL-6 and IL-8 ascites levels in ovarian cancer patients. BMC Cancer.

[16] Abreu M, Cabezas-Sainz P, Alonso-Alconada L (2020). Circulating Tumor Cells Characterization Revealed TIMP1 as a Potential Therapeutic Target in Ovarian Cancer. Cells.

[17] Mahner S, Woelber L, Eulenburg C (2010). TIMP-1 and VEGF-165 serum concentration during first-line therapy of ovarian cancer patients. BMC Cancer.

[18] Horikawa N, Abiko K, Matsumura N (2020). Anti-VEGF therapy resistance in ovarian cancer is caused by GM-CSF-induced myeloid-derived suppressor cell recruitment. Br J Cancer.

[19] Leffers N, Gooden MJM, de Jong RA (2009). Prognostic significance of tumor-infiltrating T-lymphocytes in primary and metastatic lesions of advanced stage ovarian cancer. Cancer Immunol Immunother.

[20] Zhao L, Ji G, Le X (2017). Long noncoding RNA LINC00092 acts in cancer-associated fibroblasts to drive glycolysis and progression of ovarian cancer. Cancer Res.

[21] Sang L, Ju H, Liu G (2018). LncRNA CamK-A Regulates Ca2+-Signaling-Mediated Tumor Microenvironment Remodeling. Mol Cell.

[22] Gokhale NA, Zaremba A, Shears SB (2011). Receptor-dependent compartmentalization of PPIP5K1, a kinase with a cryptic polyphosphoinositide binding domain. Biochemical Journal.

[23] Fridy PC, Otto JC, Dollins DE (2007). Cloning and characterization of two human VIP1-like inositol hexakisphosphate and diphosphoinositol pentakisphosphate kinases. J Biol Chem.

[24] Choi JH, Williams J, Cho J (2007). Purification, sequencing, and molecular identification of a mammalian PP-InsP5 kinase that is activated when cells are exposed to hyperosmotic stress. Journal of Biological Chemistry.

[25] Huyghe JR, Jackson AU, Fogarty MP (2013). Exome array analysis identifies novel loci and low-frequency variants for insulin processing and secretion. Nat Genet.

[26] Cao C H, Ling H, Han K (2021). PPIP5K2 promotes colorectal carcinoma pathogenesis through facilitating DNA homologous recombination repair. Oncogene.

[27] Chen H, Sun X, Ge W (2016). A seven-gene signature predicts overall survival of patients with colorectal cancer. Oncotarget.

[28] Han K, Wang FW, Cao CH (2020). CircLONP2 enhances colorectal carcinoma invasion and metastasis through modulating the maturation and exosomal dissemination of microRNA-17. Molecular cancer.

[29] Gu C, Nguyen H N, Ganini D (2017). KO of 5-InsP7 kinase activity transforms the HCT116 colon cancer cell line into a hypermetabolic, growth-inhibited phenotype[J]. Proceedings of the National Academy of Sciences.

[30] Machkalyan G, Hèbert T E, Miller G J (2016). PPIP5K1 suppresses etoposide-triggered apoptosis. Journal of Molecular Signaling.

[31] Limagne E, Euvrard R, Thibaudin M (2016). Accumulation of MDSC and Th17 cells in patients with metastatic colorectal cancer predicts the efficacy of a FOLFOX-bevacizumab drug treatment regimen. Cancer Res.

[32] Obermajer N, Muthuswamy R, Lesnock J (2011). Positive feedback between PGE2 and COX2 redirects the differentiation of human dendritic cells toward stable myeloid-derived suppressor cells. Blood.

[33] Hernando JJ, Park T-W, Fischer H-P (2007). Vaccination with dendritic cells transfected with mRNA-encoded folate-receptor-α for relapsed metastatic ovarian cancer. Lancet Oncol.

[34] Wu L, Deng Z, Peng Y (2017). Ascites-derived IL-6 and IL-10 synergistically expand CD14(+)HLA-DR(-/low) myeloid-derived suppressor cells in ovarian cancer patients. Oncotarget.

[35] Taki M, Abiko K, Baba T (2018). Snail promotes ovarian cancer progression by recruiting myeloid-derived suppressor cells via CXCR2 ligand upregulation. Nat Commun.

[36] Mutch DG, Prat J (2014). 2014 FIGO staging for ovarian, fallopian tube and peritoneal cancer. Gynecol Oncol.

[37] Huang DW, Sherman BT, Lempicki RA (2009). Systematic and integrative analysis of large gene lists using DAVID bioinformatics resources. Nat Protoc.

[38] Huang DW, Sherman BT, Lempicki RA (2009). Bioinformatics enrichment tools: paths toward the comprehensive functional analysis of large gene lists. Nucleic Acids Res.

[39] Lin H, Fridy PC, Ribeiro AA (2009). Structural analysis and detection of biological inositol pyrophosphates reveal that the family of VIP/diphosphoinositol pentakisphosphate kinases are 1/3-kinases. J Biol Chem.

[40] Jackson CL (2009). Mechanisms of transport through the Golgi complex. J Cell Sci.

[41] Gleeson PA, Anderson TJ, Stow JL (1996). p230 is associated with vesicles budding from the trans-Golgi network. J Cell Sci.

[42] Egea G, Lázaro-Diéguez F, Vilella M (2006). Actin dynamics at the Golgi complex in mammalian cells. Curr Opin Cell Biol.

[43] Halberg N, Sengelaub CA, Navrazhina K (2016). Drive Malignant Secretion Article PITPNC1 Recruits RAB1B to the Golgi Network to Drive Malignant Secretion. Cancer Cell.

[44] Dippold HC, Ng MM, Farber-Katz SE (2009). GOLPH3 bridges phosphatidylinositol-4- phosphate and actomyosin to stretch and shape the Golgi to promote budding. Cell.

[45] Dunkelberger J R, Song W C (2010). Complement and its role in innate and adaptive immune responses. Cell research.

[46] Szklarczyk D, Franceschini A, Wyder S (2015). STRING v10: protein-protein interaction networks, integrated over the tree of life. Nucleic Acids Res.

[47] Nadeem L, Munir S, Fu G (2011). Nodal signals through activin receptor-like kinase 7 to inhibit trophoblast migration and invasion: implication in the pathogenesis of preeclampsia. Am J Pathol.

[48] Gasson JC, Golde DW, Kaufman SE (1985). Molecular characterization and expression of the gene encoding human erythroid-potentiating activity. Nature.

[49] Bi S, Hong PW, Lee B, Baum LG (2011). Galectin-9 binding to cell surface protein disulfide isomerase regulates the redox environment to enhance T-cell migration and HIV entry. Proc Natl Acad Sci U S A.

[50] Bax D V, Bernard SE, Lomas A (2003). Cell adhesion to fibrillin-1 molecules and microfibrils is mediated by alpha 5 beta 1 and alpha v beta 3 integrins. J Biol Chem.

[51] Jovanović J, Takagi J, Choulier L (2007). αvβ6 is a novel receptor for human fibrillin-1: comparative studies of molecular determinants underlying integrin-RGD affinity and specificity. J Biol Chem.

[52] Javitt G, Grossman-Haham I, Alon A (2019). cis-Proline mutants of quiescin sulfhydryl oxidase 1 with altered redox properties undermine extracellular matrix integrity and cell adhesion in fibroblast cultures. Protein Sci.

[53] Surace L, Lysenko V, Surace L (2015). Complement Is a Central Mediator of Radiotherapy- Induced Tumor-Specific Immunity and Clinical Article Complement Is a Central Mediator of Radiotherapy-Induced Tumor-Specific Immunity and Clinical Response. Immunity.

[54] Janát-Amsbury MM, Yockman JW, Anderson ML (2006). Comparison of ID8 MOSE and VEGF-modified ID8 cell lines in an immunocompetent animal model for human ovarian cancer. Anticancer Res.

[55] Whiteside TL (2008). The tumor microenvironment and its role in promoting tumor growth. Oncogene.

[56] Wright AA, Bohlke K, Armstrong DK (2016). Neoadjuvant chemotherapy for newly diagnosed, advanced ovarian cancer: Society of Gynecologic Oncology and American Society of Clinical Oncology Clinical Practice Guideline. Gynecol Oncol.

[57] Bekker P, Dairaghi D, Seitz L (2016). Characterization of pharmacologic and pharmacokinetic properties of CCX168, a potent and selective orally administered complement 5a receptor inhibitor, based on preclinical evaluation and randomized phase 1 clinical study. PLoS One.

[58] Jayne DRW, Merkel PA, Schall TJ, Bekker P (2021). Avacopan for the treatment of ANCA-associated vasculitis. N Engl J Med.

[59] Shi M, He J (2016). ColoFinder: a prognostic 9-gene signature improves prognosis for 871 stage II and III colorectal cancer patients. PeerJ.

[60] Ohtsuka M, Ling H, Ivan C (2016). H19 Noncoding RNA, an Independent Prognostic Factor, Regulates Essential Rb-E2F and CDK8-β-Catenin Signaling in Colorectal Cancer. EBioMedicine.

[61] Howley B (2018). V, Link, L. A, Grelet, S, El-Sabban, M. & Howe, P. H. A CREB3-regulated ER-Golgi trafficking signature promotes metastatic progression in breast cancer. Oncogene.

[62] Riihilä P, Viiklepp K, Nissinen L (2020). Tumour-cell-derived complement components C1r and C1s promote growth of cutaneous squamous cell carcinoma. Br J Dermatol.

[63] Markiewski MM, DeAngelis RA, Benencia F (2008). Modulation of the antitumor immune response by complement. Nat Immunol.

[64] Zhou F, Wan Q, Lu J (2019). Pim1 Impacts Enterovirus A71 Replication and Represents a Potential Target in Antiviral Therapy. iScience.

[65] Dong L, Dong Q, Chen Y (2018). Novel HDAC5-interacting motifs of Tbx3 are essential for the suppression of E-cadherin expression and for the promotion of metastasis in hepatocellular carcinoma. Signal Transduct Target Ther.

[66] Bi H, Yang X, Yuan J (2013). H19 inhibits RNA polymerase II-mediated transcription by disrupting the hnRNP U - actin complex. BBA - Gen Subj.

