# Supplementary material for: Suppressing MDSC Infiltration in Tumor Microenvironment Serves as an Option for Treating Ovarian Cancer Metastasis
Source: Int J Biol Sci. 2022 May 21;18(9):3697–713. doi: 10.7150/ijbs.70013 (PMC9254473; doi:10.7150/ijbs.70013)
Supplement: Supplementary file 1 — Supplementary figures. [file ijbsv18p3697s1.pdf]

Supplemental information

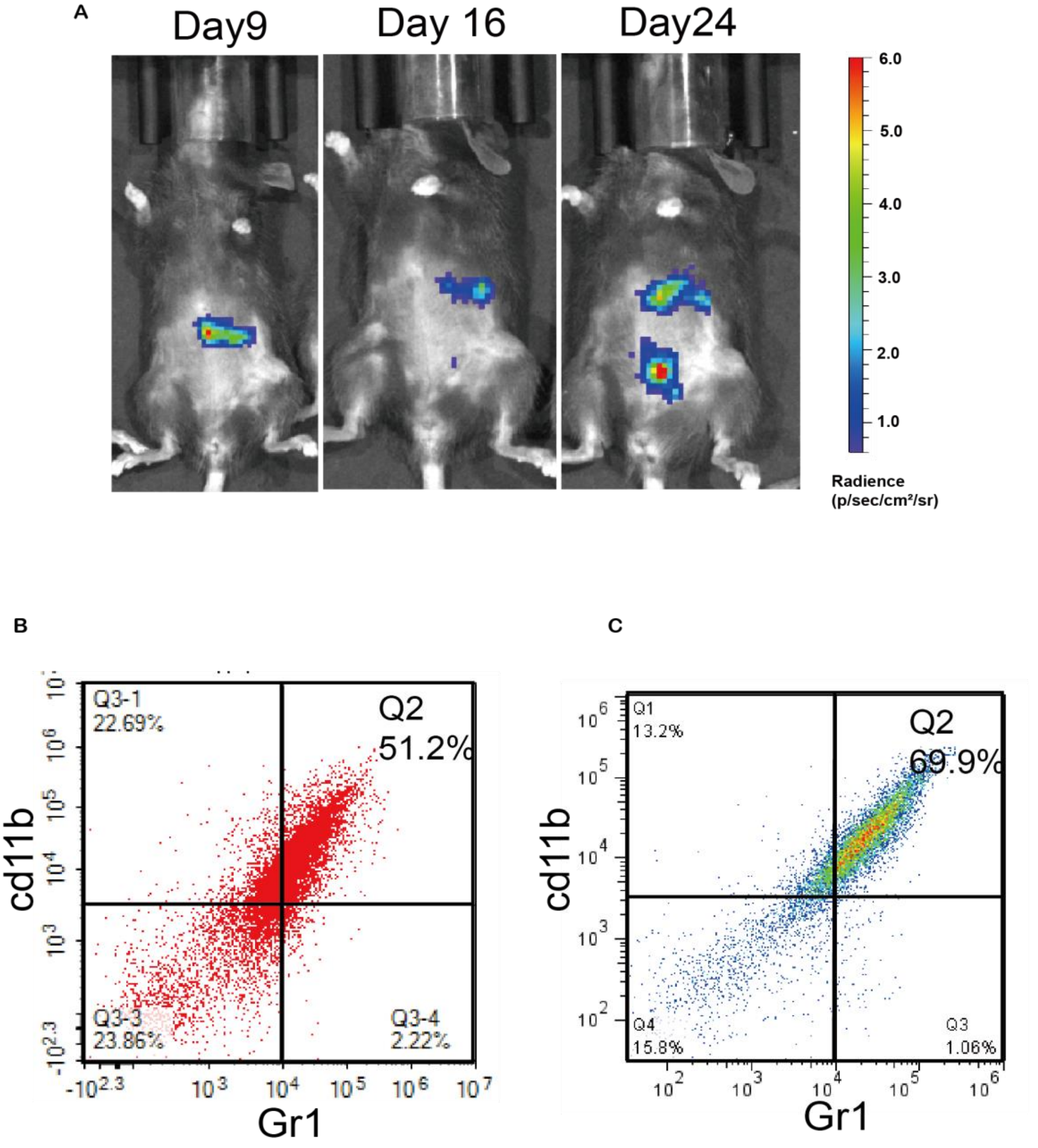

Fig.S1, related to Figure 1, MDSC infiltration in mice TME.

A. Bioluminescence imaging of ID8-luciferase cells. ID8-luciferase cells (5x105) were inoculated i.p. into 6-week C57BL/6 mice (n=6 per group).

B. Flow cytometry analysis of MDSCs in mice TME. After 9 days, tumors were collected from ID8-luciferase cells (5x106) inoculated s.c. into 6-week C57BL/6 mice (n=6 per group).

C. Flow cytometry analysis of MDSCs in mice TME. After 15 days, tumors were collected from ID8 cells (1x107) inoculated s.c. into 6-week C57BL/6 mice (n=6 per group).

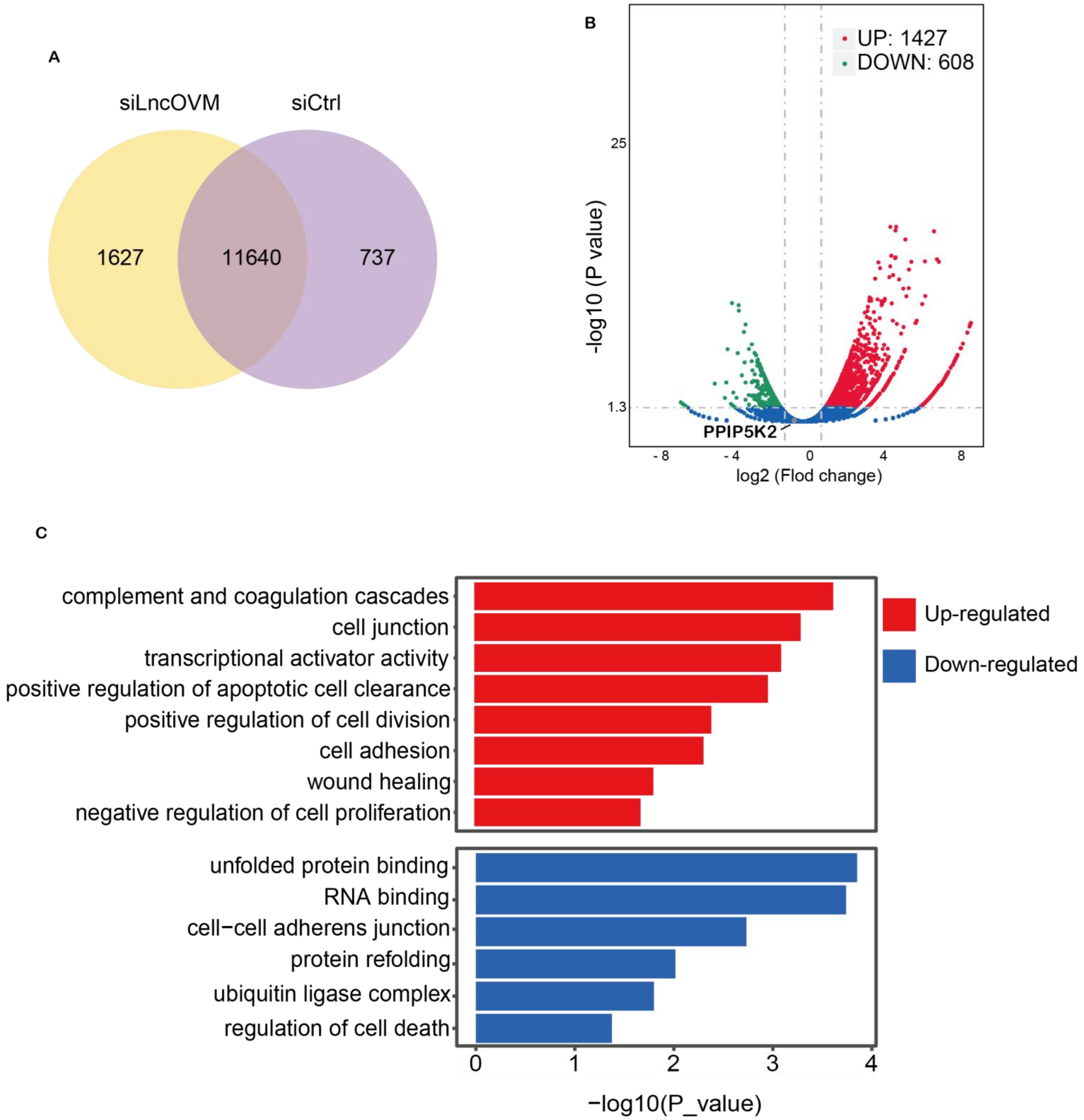

Fig.S2, related to Figure 2, RNA-Seq analysis implies the differential expression in LncOVM depletion and normal cells.

A. The venn diagram of all RNA-Seq results in A2780s cells.

B. The volcano plot representation of differential expression analysis of genes in the siLncOVM versus the control (siCtrl) for A2780s RNA-seq data sets. Red and green points mark the genes with significantly increased or decreased expression respectively (FDR<0.01). Dots above the dashed line represent proteins for which differences were significant.

C. The plot representation of up-regulated (red) and down-regulated (blue) molecular functions and pathways in the siLncOVM group compared to control group (siCtrl) for A2780s RNA-seq data sets. The functional annotation of regulated genes performed by DAVID analysis tools. The down-regulated group was defined as the ratio of siLncOVM/siNC ≤0.4, while the up-regulated group was defined as the ratio ≥5.

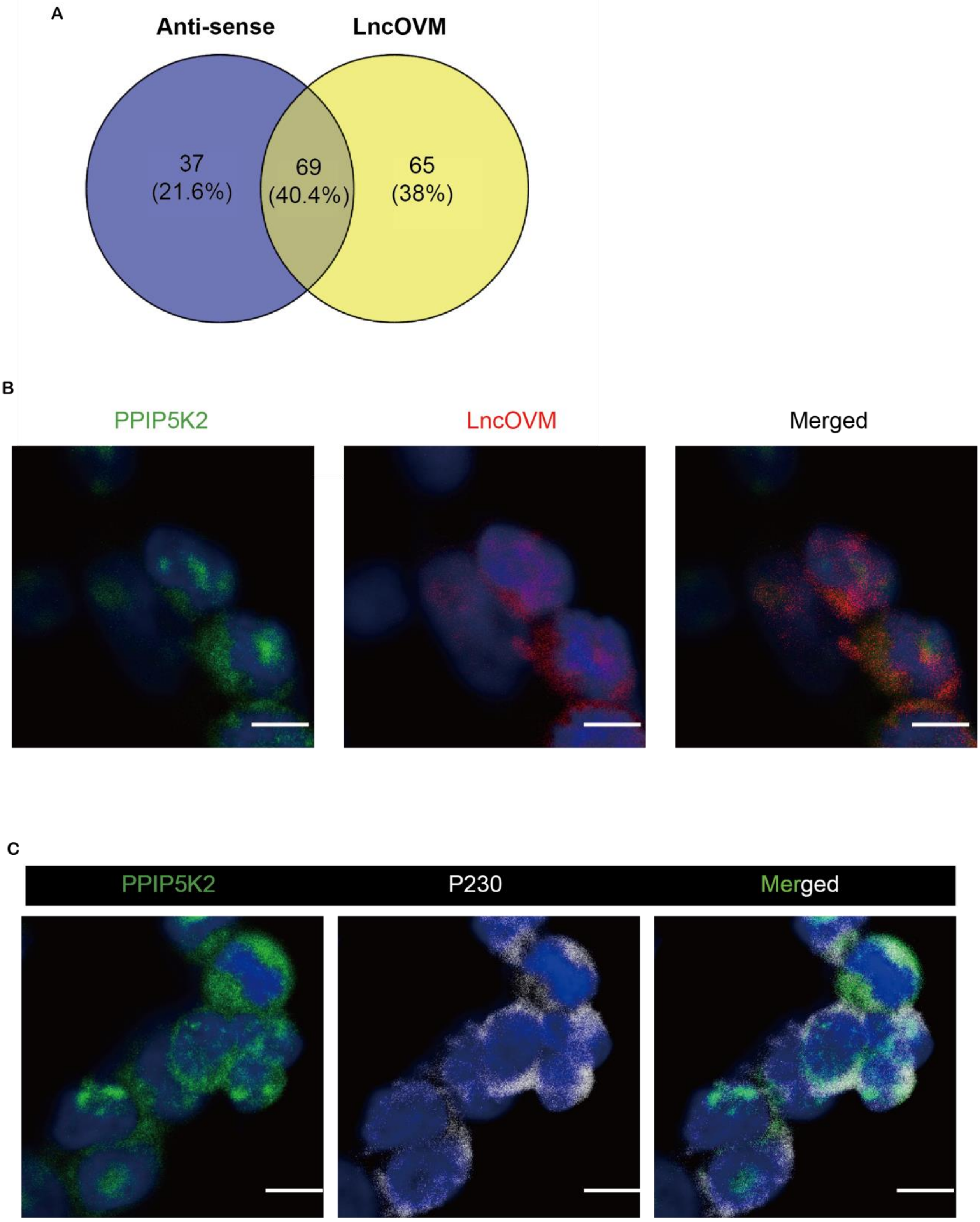

Figure S3, related to Figure 3, LncOVM complexes with protein PPIP5K2.

A. LncOVM or its antisense pull-down proteins identified by mass spectrometry. 65 proteins (yellow) were candidate proteins.

B. Co-localization of PPIP5K2 and LncOVM detected by Immunofluorescence image in 293T cells. LncOVM was hybridized with oligonucleotide probes with Cy3 fluorophore.

C. Immunofluorescence analysis of PPIP5K2 and Golgi marker P230 in 293T cells.

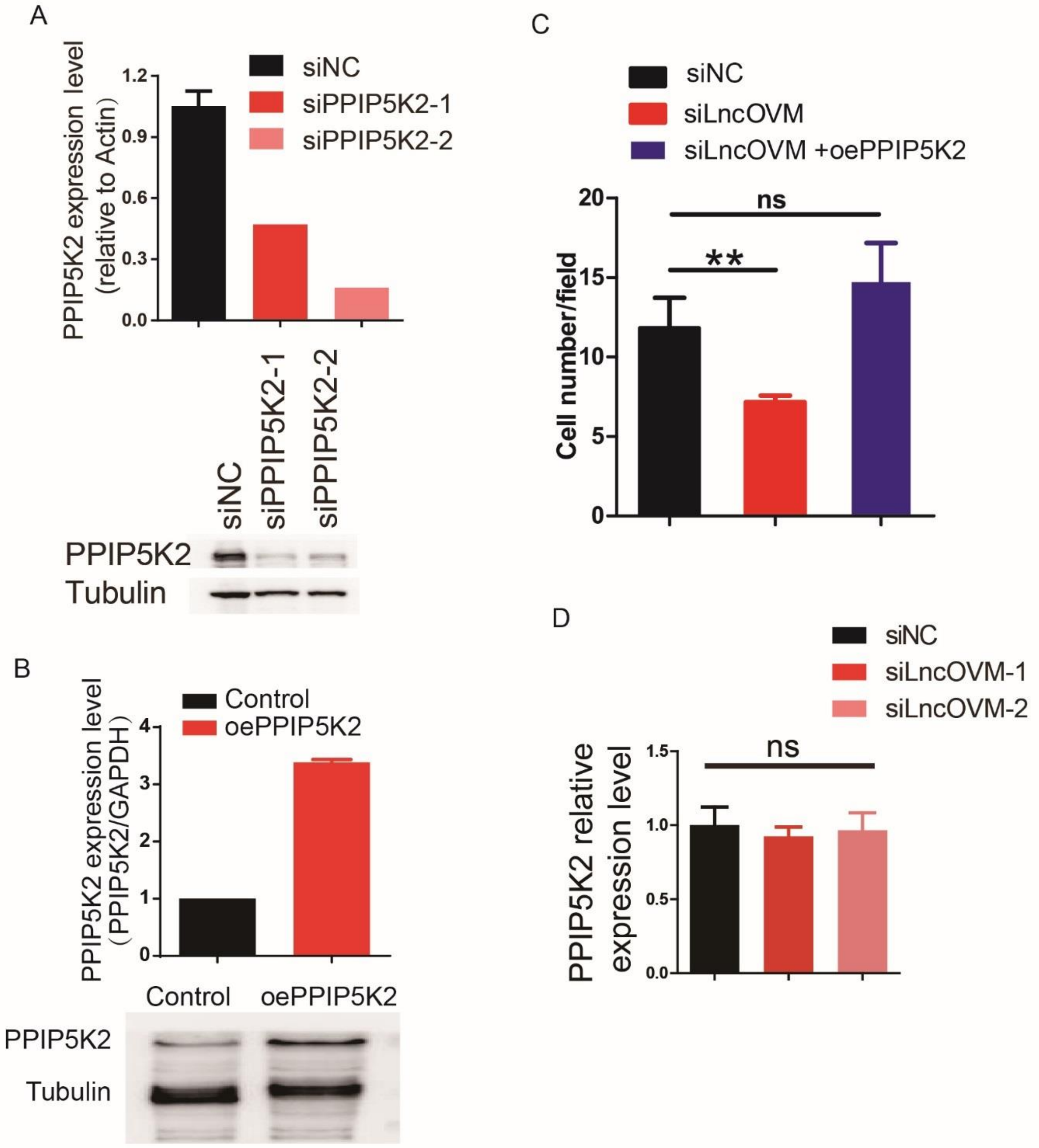

Fig.S4, related to Figure 3, LncOVM complexes with protein PPIP5K2 which promotes ovarian cancer progression.

A. RT-PCR analysis of PPIP5K2 genes expression and Western Blot of proteins in A2780S cells with negative control siRNA (siNC) and two PPIP5K2 siRNAs (siPPIP5K2-1/2).

B. Western blot of PPIP5K2 protein and RT-PCR analysis of PPIP5K2 genes expression in A2780S cells with negative control vector and PPIP5K2 overexpression.

C. Colony formation of A2780s cells.

D. RT-PCR analysis of PPIP5K2 RNA expression level with two LncOVM siRNAs and siNC.

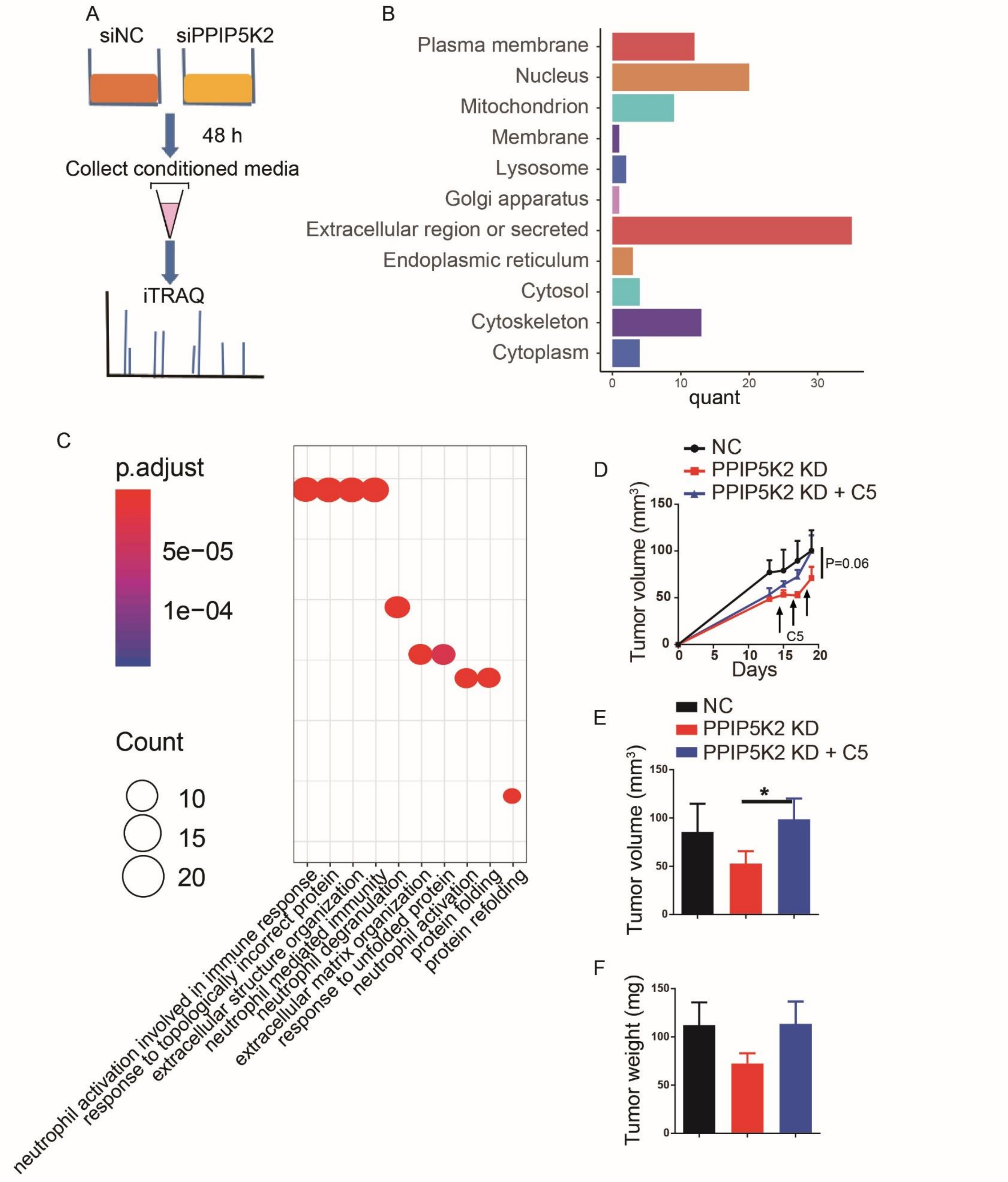

Fig.S5, related to Figure 5, Secretion protein analysis by iTRAQ.

A. Experimental scheme for identification of secretion proteins by iTRAQ. Conditioned medium was collected from 48-hour starved A2780S control and PPIP5K2 knockdown cells.

B. Protein location of iTRAQ identified proteins.

C. The top 10 enrichments of gene function according to the protein interaction network in iTRAQ data. Analyze on the Gene Ontology Resource (http://geneontology.org/).

D-F. Tumor growth curves, and post-dissection measured tumor weight and volume in subcutaneous inoculation C57BL/6 mice model. The mouse cell line ID8 control cells (1x107) and PPIP5K2 knock-down (KD) cells were inoculated s.c. into 6-week C57BL/6 mice. Complement C5 (0.025 mg/kg) was injected intraperitoneally every second day from the 14th day. (n=5).

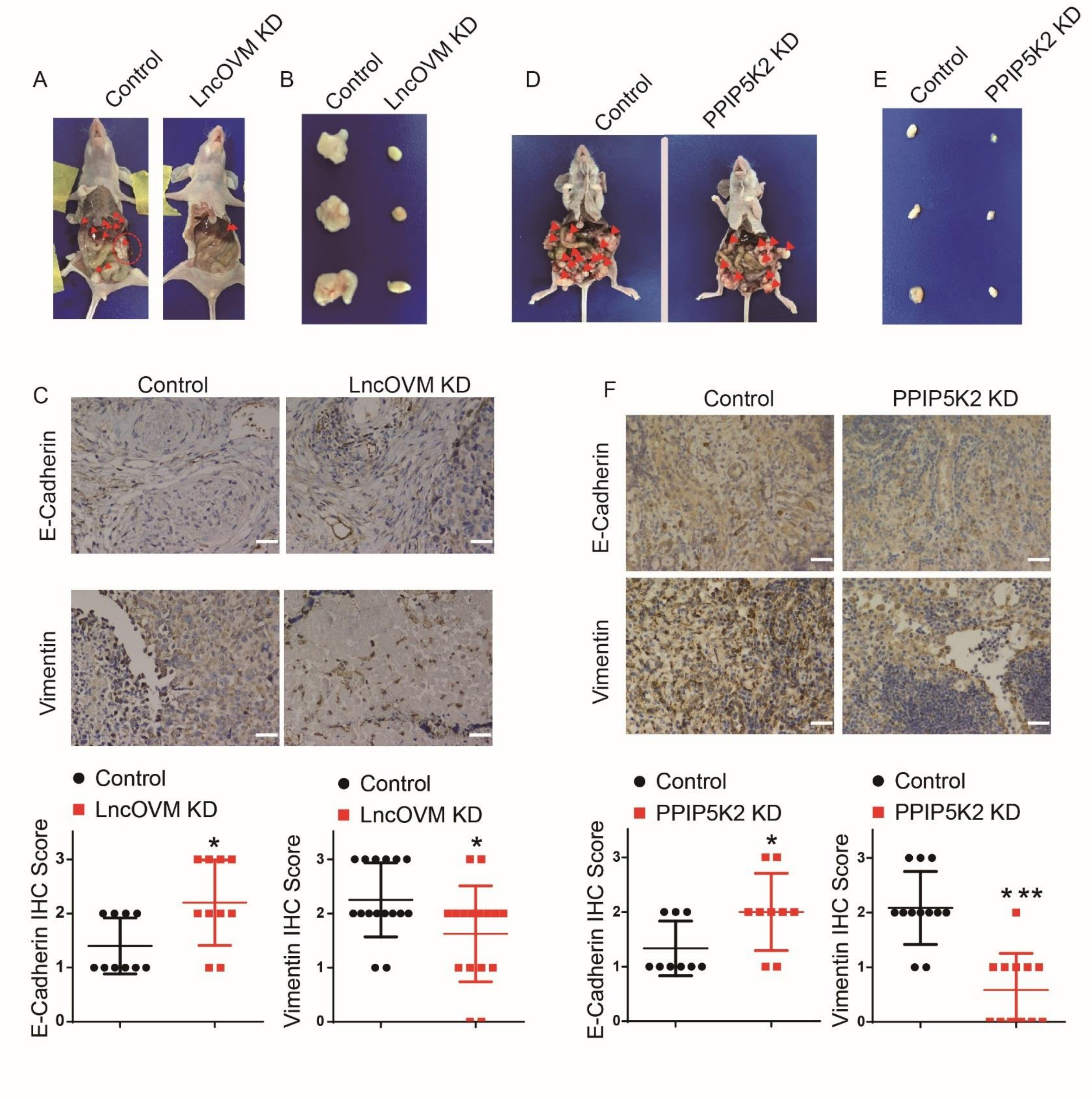

Fig.S6, related to Figure 6, Secretion protein complement C5 impacts MDSC recruitment.

A. Photo of balb/c nude mice with metastatic nodules in the abdominal cavities of an intraperitoneally Inoculation model. A2780S control cells and LncOVM knock-down (KD) cells (5x106) were injected i.p. into 6-week balb/c nude mice (n=5).

B. Photo of A2780S tumors with control cells and LncOVM KD cells (1x107) inoculated s.c. into 6-week balb/c nude mice (n=5).

C. IHC staining indicated expression of E-cadherin and vimentin in A2780s WT and LncOVM depleted tumors. The staining scores were quantified in right plot.

D. Photo of balb/c nude mice with metastatic nodules in the abdominal cavities of an intraperitoneally inoculation model. A2780S control cells and PPIP5K2 knock-down (KD) cells (5x106) were injected i.p. into 6-week balb/c nude mice (n=5).

E. Photo of A2780S tumors with control cells and PPIP5K2 KD cells (1x107) inoculated s.c. into 6-week balb/c nude mice (n=5).

F. IHC staining indicated expression of E-cadherin and vimentin in A2780s WT and PPIP5K2 depleted tumors. The staining scores were quantified in right plot.

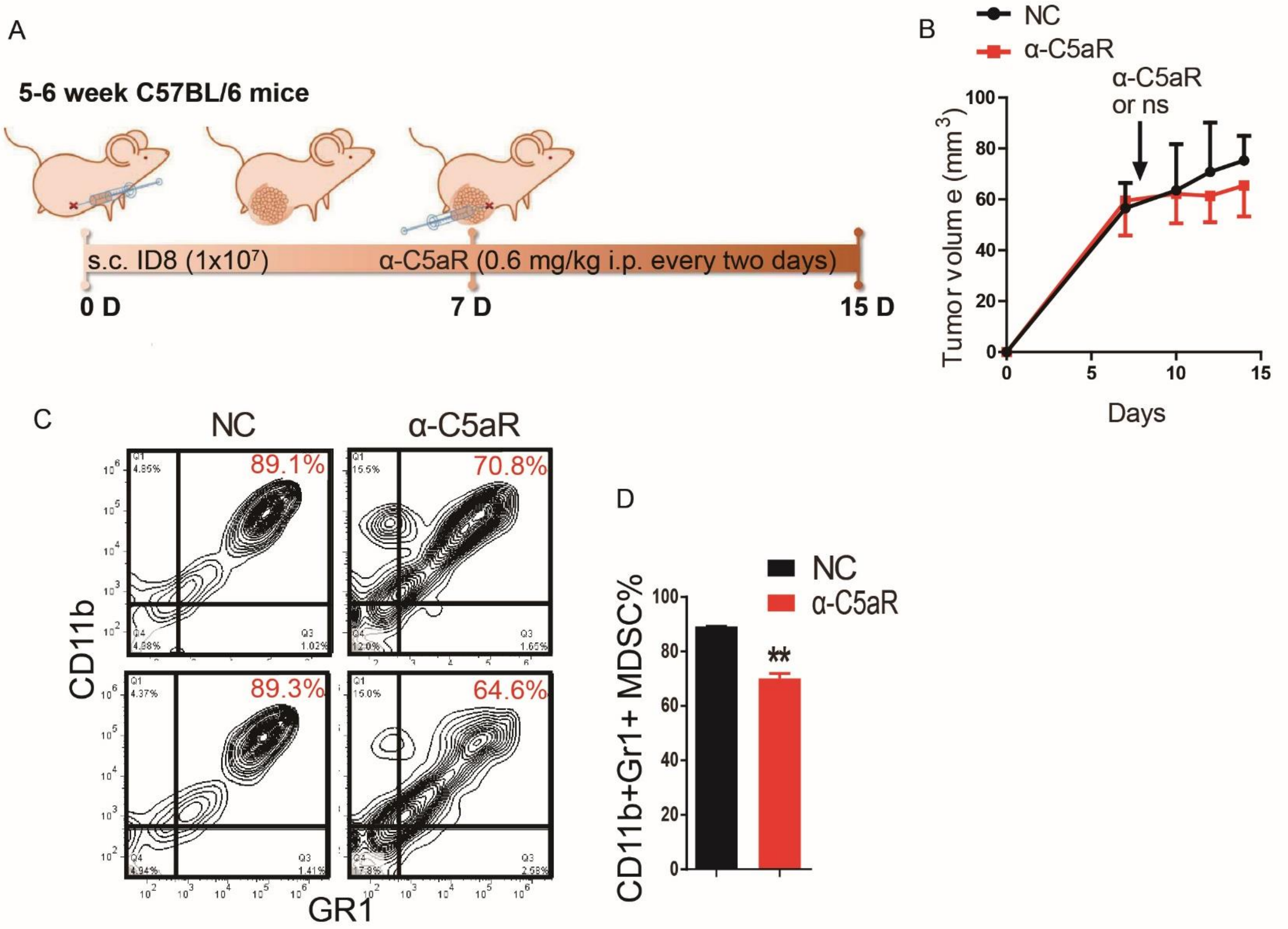

Fig.S7, related to Figure 7, Targeting LncOVM-PPIP5K2-Complement C5 axis suppresses MDSC infiltration in TME against cancer metastasis and tumorigenesis

A. Experimental scheme for C57BL/6 mice treated with C5aR antibody (α-C5aR) or normal saline as control by injecting into tumor surrounding surface. ID8 cells (1x107) were inoculated s.c. into 6-week C57BL/6 mice (n=6 per group).

The anti-C5aR antibody (α-C5aR) or normal saline as negative control (NC) was injected into tumor surrounding surface (0.6 mg/kg) every second day.

B. Tumor growth curves of the C57BL/6 mice treated with C5aR antibody (α-C5aR) or normal saline.

C-D. The flow cytometry showed fraction change of MDSC (CD11b+, Gr-1+) in ID8 mice tumor microenvironment.
